# Nanodot‐Inspired Precise Bacterial Gene Suppression in a Smart Hydrogel Bandage for Underwater Wound Healing

**DOI:** 10.1002/advs.202415169

**Published:** 2025-02-14

**Authors:** Qingsong Zhang, Menghan Lu, Richang Ou, Hong Lin, Guanhua Xuan, Xiudan Wang, Xiaofeng Xu, Weiwei Zhang, Guoqing Wang

**Affiliations:** ^1^ MOE Key Laboratory of Evolution & Marine Biodiversity and Institute of Evolution & Marine Biodiversity Ocean University of China 5 Yushan Road Qingdao 266003 China; ^2^ Laboratory for Marine Drugs and Bioproducts Qingdao Marine Science and Technology Center Qingdao 266237 China; ^3^ SKL of Marine Food Processing & Safety Control College of Food Science and Engineering Ocean University of China 1299 Sansha Road Qingdao 266404 China; ^4^ College of Materials Science and Engineering Ocean University of China Qingdao 266100 China; ^5^ School of Marine Sciences Ningbo University 169 Qixingnan Road Ningbo 315832 China

**Keywords:** antisense oligonucleotide, carbon dot, gene regulation, nucleic acid delivery, underwater wound treatment

## Abstract

The complex and dynamic nature of aquatic ecosystems, particularly in marine environments, makes managing wound infections a significant challenge for individuals engaged in underwater activities and for aquatic organisms. Although antibiotics have played a critical role in safeguarding humans and aquatic health, their risk of drug resistance and environmental impact present substantial obstacles to long‐term sustainability. Using fin rot disease in turbot (*Scophthalmus maximus*) caused by infection of *Vibrio anguillarum* (*V. anguillarum*) as a model, a new strategy is presented that employs a carbon dot (CD)‐based antisense oligonucleotide (ASO) delivery system, combined with an adhesive hydrogel, to achieve targeted gene silencing of *V. anguillarum* for underwater healing. The CDs that cause enhanced cytoplasmic membrane permeability, efficiently deliver ASOs into *V. anguillarum* without requiring additional equipment or chemical facilitators. The specific design of the ASO sequence enables targeted silencing of *empA*, achieving efficiency as high as 71.2%. An adhesive hydrogel is applied to boost the local concentration of ASO/CDs at wound sites in seawater, effectively sealing the infected area and preventing fin rot disease in turbot. This study pioneer targeted bacterial gene regulation using CD‐based delivery integrated with a hydrogel bandage, offering practical solutions for managing underwater bacterial diseases.

## Graphical abstract

This study presents a conceptually new approach to underwater wound healing. By exploiting carbon dot (CD)‐delivered specific antisense oligonucleotide (ASO) to bacteria, targeted pathogenic gene suppression is achieved. Combined with an adhesive hydrogel, the ASO/CDs affords localized treatment of pathogen‐infected wounds underwater. This approach offers an innovative, sustainable solution for managing human and organism diseases in a submerged environment.

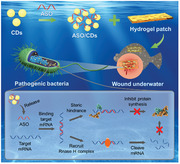

## 1 Introduction

Bacterial infections in underwater environments pose significant challenges to the health of both human and aquatic organisms.^[^ ^1^, ^2^ ^]^ In human health, managing bacterial infections in wounds that remain submerged, such as during bathing or swimming, is notoriously difficult due to the constant exposure to water, which washes away conventional treatments and impedes effective drug delivery.^[^ ^3^, ^4^ ^]^ In aquatic ecosystems, similarly, bacteria can colonize open wounds of marine organisms, often leading to severe infections, impaired healing, and in some cases, large‐scale mortality.^[^ ^5^, ^6^ ^]^ Traditionally, antibiotics have been the mainstay of disease prevention and treatment.^[^ ^7^, ^8^ ^]^ However, their application in underwater conditions faces several limitations. Antibiotics often fail to remain localized, leading to environmental contamination and contributing to the rise of antibiotic‐resistant bacterial strains.^[^ ^9^ ^]^ This is also the case for the emerging antibacterial nanomaterials that also lack specificity and targeting capability in underwater treatment.^[^ ^10^, ^11^, ^12^ ^]^ These issues are compounded by concerns over drug residues that affect the broader aquatic environment. Consequently, developing an innovative approach that overcomes the limitations and enables targeted wound treatment in submerged environments has remained an open challenge for microbiologists and marine biotechnologists.

Gene silencing based on the use of antisense oligonucleotide (ASO), presents a promising alternative for targeted bacterial gene regulation.^[^ ^13^, ^14^ ^]^ Nevertheless, the application in underwater contexts has been limited by challenges in effectively delivering nucleic acids into bacterial cells, especially in harsh aquatic environments. Traditional delivery methods, such as electroporation and chemical transformation, are strictly dependent on specialized operation in a qualified laboratory.^[^ ^15^, ^16^ ^]^ They are either ineffective in water or necessitate complex and impractical apparatuses, limiting the potential for localized bacterial gene regulation in submerged environments. Although nanoparticles, such as gold and silica nanoparticles, have proven effective in delivering various cargoes into eukaryotic cells via endocytosis,^[^ ^17^, ^18^, ^19^ ^]^ the delivery of nucleic acids to bacteria has been greatly hindered by the rigid bacterial cell walls and their limited membrane permeability.^[^ ^20^ ^]^ Further, the dynamic nature of aquatic ecosystems introduces further obstacles. The presence of water currents, varying temperatures, and diverse microbial communities can impact the local concentration and thereby the efficacy of ASOs, making consistent delivery a significant hurdle. Although some biopesticides (e.g., RNA) have been proposed as an innovative approach for combatting underwater bacterial infection, their practical implementation has mainly encountered problems of swift environmental degradation and limited efficacy.^[^ ^21^ ^]^ As a result, there is also a pressing need for innovative delivery systems that can enhance the uptake of ASOs by bacterial cells while maintaining their local concentration in water.

**Figure 1:**
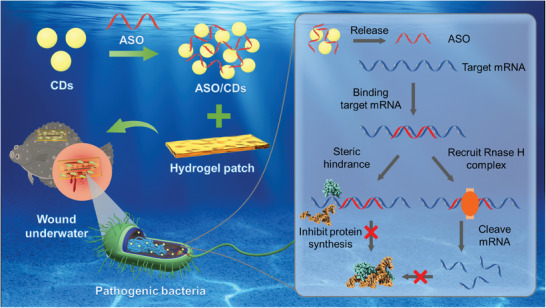
Schematic illustration of the CDs‐based ASO delivery system assisted with an underwater adhesion hydrogel for bacterial disease control for wound treatment in submerged environments. The ASO for targeting a vir gene in the pathogen is loaded and delivered into bacteria infecting the wound by the CDs, followed by its release from the CDs in the bacteria and binding with the complementary mRNA of a target gene. This process results in the inhibition of the expression of the target gene through steric hindrance and/or RNase H‐mediated mRNA cleavage, preventing the synthesis of pathogenic protein and thereby the occurrence of bacterial disease. The efficiency of the ASO/CDs in disease prevention and therapy can be enhanced by an underwater adhesive hydrogel.

Recently, carbon dots (CDs) with excellent biocompatibility have emerged as popular carriers for nucleic acid delivery to mammalian cells for cancer therapy.^[^ ^22^, ^23^ ^]^ Compared with noble metal nanoparticle carriers, furthermore, CDs often have smaller size and weak antimicrobial activity.^[^ ^11^, ^24^ ^]^ It has been reported that their minimal bacterial inhibition concentration is higher than silver nanoparticles by 1–2 orders of magnitude.^[^ ^25^, ^26^, ^27^ ^]^ The above features of CDs suggest the potential for ASO delivery to bacterial cells. In this study, we address the challenges of ASO delivery into pathogenic bacteria for wound care in water by developing a conceptually new method that integrates the CD‐mediated delivery of specific ASO with the localization capability of an adhesive hydrogel (**Figure** **1**). We demonstrate that the CDs that have a diameter of ≈3 nm possess excellent membrane‐permeating properties while exerting negligible influence on bacterial activity, enabling them to transport ASO into bacterial cells without the need for additional chemical facilitators or devices. By using the *empA* gene of *V. anguillarum* as the target, we demonstrate efficient gene silencing with up to 71.2% inactivation, using the CDs as the ASO‐delivery vector. The combination with an underwater adhesive hydrogel allows for localized, sustained release of the ASO/CD complexes at the infection site, ensuring prolonged therapeutic action and effectively preventing fin rot disease in turbot. This study paves the way for underwater bacterial disease management, with potential application in both human and aquatic health.

## 2 Experimental Section

2.1

Citric acid (CA, ≥ 99.5%) and o‐nitro‐phenyl‐*β*‐*D*‐galactopyranoside (ONPG, 99%), tannic acid (TA, 98%), chitosan (MW = 150000), acrylic acid (AA, >99%), *N*,*N′*‐methylenebisacrylamide, and ammonium persulfate (APS, 98.5%) were obtained from Macklin Biochemical Co., Ltd. (Shanghai, China). Polyethyleneimine (PEI, MW = 2000, 50% aqueous solution) and phosphate buffer saline (PBS, pH 7.4) were obtained from Yuanye Biotechnology Co., Ltd. (Shanghai, China). Cellulose membrane dialysis bags (MW 1000, MD44) were obtained from Yibo Biotechnology Co., Ltd (Hunan, China). The nucleic acid sequences were purchased from Sangong Bioengineering Co., Ltd. (Shanghai, China), which are listed in Table S1 (Supporting Information). The CCK‐8 kit was purchased from Beijing Solarbio Science & Technology Co., Ltd. (Beijing, China). Trizol reagent, HiScript III All‐in‐one RT SuperMix Perfect for qPCR, and ChamQ SYBR Color qPCR Master Mix (Low ROX Premixed) were obtained from Vazyme Biotech Co., Ltd. (Nanjing, China). Leibovitz's L‐15 Medium, Dulbecco's Modified Eagle Medium (DMEM), Fetal Bovine Serum (FBS), and Penicillin‐Streptomycin (P/S) solution were purchased from Shanghai XP Biomed Ltd. (Shanghai, China). Luria–Bertani (LB) medium, aluminum nitrate nanohydrate, and all the other reagents used in this study were purchased from Sinopharm Chemical Reagent (Shanghai, China). Ultrapure water (>18 MΩ cm, 25 ± 2 °C) from an arium pro water purification system (Sartorius, Germany) was used throughout the experiments.

The CDs were freeze‐dried using an Alpha 1–4 LDplus vacuum freeze dryer (Christ, German). The CDs were characterized by high‐resolution transmission electron microscopy (HRTEM) (JEM‐2100 Plus, JEOL, Japan). The crystal structure of the CDs was measured by X‐ray diffraction (XRD) (D8 Advance, Bruker, German). The UV–vis absorption spectra of the CDs were recorded using a UV‐1800 spectrophotometer (Shimadzu, Japan). The fluorescence spectra of the CDs and the ASO/CDs with a CD‐to‐ASO mass ratio of 20:1 was measured using an RF‐6000 spectrophotometer (Shimadzu, Japan). The elemental composition of the CDs was determined by X‐ray photoelectron spectroscopy (XPS) (ESCALAB Xi+, Thermo Fisher, USA). The chemical structures of the CDs were analyzed by a Fourier transform infrared (FTIR) spectrometer (Nicolet iS5, Thermo Fisher, USA). The surface charge of the nanoparticles in aqueous suspension was determined using a zeta potential analyzer (Nano ZS90, Malvern Instruments Inc., UK). The results of agarose gel electrophoresis were analyzed using a gel imager (Gel Doc XR+, BIO‐RAD, USA). The absorbance of the bacterial suspensions was measured at 600 nm using a microplate reader (Varioskan Flash 3001, Thermo Fisher, USA). The delivery efficiency for the ASO using the CDs was evaluated using flow cytometry (FACSVerse, Becton, Dickinson and Company, USA). The internalization of the ASO/CDs by bacteria and staining with Hoechst 33342 were observed using a fluorescence microscope (Ni‐E, NIKON, Japan). The values of Ct were determined using a real‐time fluorescent quantitative PCR instrument (qPCR) (PikoReal real‐time PCR, Thermo, USA). The mechanical and adhesive properties of the hydrogel were measured using a motorized testing stand (ESM303, Mark‐10, USA).

### 2.2 Synthesis of the CDs

**Figure 2:**
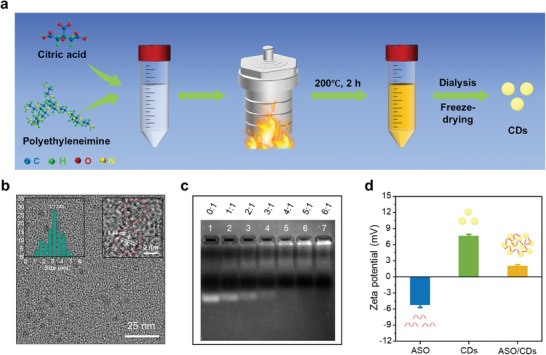
Synthesis and characterizations of the CDs as the carrier of the ASO. a) Schematic illustration of the one‐step hydrothermal synthesis of the CDs. b) A representative TEM image of the CDs. The insets show the histogram of the size distribution (left) and the HRTEM image (right) of the CDs. c) Agarose gel (1%) electrophoresis for testing the loading capacity of the CDs for the ASO (116 ng) at different mass ratios. d) Zeta potential measurement results for the ASO, CDs, and ASO/CDs. The mass ratio of the CDs to the ASO in the ASO/CDs is 5:1.

The CDs were synthesized by a facile one‐step hydrothermal method using CA and PEI as the precursors, as schematically illustrated in **Figure** **2**a.^[^ ^11^ ^]^ Briefly, CA (1.0 g) and PEI (2.0 g) were dissolved in water (10 mL) and sonicated for 10 min. Then, the mixture was transferred into a Teflon‐lined hydrothermal reactor and carbonized at 200 °C for 2 h. After cooling to room temperature, the yellow solution was dialyzed against water for 24 h using a dialysis bag with a molecular weight cutoff of 1000 Da. Finally, the dialysate was lyophilized to yield a yellow powder of the CDs.

### 2.3 ASO Loading on the CDs

The ASO (116 ng) was incubated with the CDs at various mass ratios (1:1, 1:2, 1:3, 1:4, 1:5, and 1:6) in ultrapure water for 30 min. Each resultant complex was characterized using 1% agarose gel electrophoresis. Band intensity was detected with a gel imager to evaluate the efficiency of the ASO loading onto the CDs. Additionally, the surface charges of the CDs, ASO, ASO, and CDs complexes (ASO/CDs) were determined.

### 2.4 Bacterial Strains and Culture Conditions

*V. anguillarum* NBRC 13266 was cultured in an LB medium containing 2% sodium chloride at 28 °C. The bacterial cell concentration was determined by measuring the absorbance of the suspension at 600 nm. For the *V. anguillarum* used in the present study, 1 OD_600_ of the bacterial suspension corresponds to a concentration of 4.2 × 10^9^ CFU mL^−1^.

### 2.5 Fluorescent Localization of the ASO

*V. anguillarum* was labeled with Hoechst 33342 dye. The effect of the CDs on ASO delivery was determined using fluorescence microscopy. Briefly, Cy3‐ASO (10 µM, 50 µL) was incubated with the CDs (1.16 mg mL^−1^, 50 µL) for 30 min, and the mixture (100 µL) was then mixed with *V. anguillarum* suspension (900 µL, 10^6^ CFU mL^−1^) and incubated in a shaking incubator (28 °C, 180 rpm) for 2 h. The final concentration of the Cy3‐ASO in the system (1 mL) was 500 nm, and the final concentration of the CDs was 58 µg mL^−1^. The bacterial suspension was then subjected to three centrifugation/washing cycles with PBS containing 0.1% Triton X‐100 and was then resuspended in PBS (100 µL). Afterward, Hoechst 33342 dye (100 µL, 10 µg mL^−1^) was added to the bacteria and incubated (28 °C, 180 rpm) in the dark for 30 min, followed by three washes with PBS. Smears were prepared and observed under a fluorescence microscope. Experiments conducted with the CDs alone and the Cy3‐ASO alone were used as controls.

### 2.6 Bacterial Internalization and Optimization Conditions of Cy3‐ASO

The Cy3‐ASO (10 µM, 50 µL) was incubated with the CDs (1.16 mg mL^−1^, 50 µL) for 30 min. To optimize incubation time, the mixture (100 µL) was cultured with *V. anguillarum* at a density of 10^6^ CFU mL^−1^ in LB liquid medium (900 µL) at 28 °C for varying durations (0, 0.5, 1, 2, 3 and 5 h). The final concentration of the Cy3‐ASO was 500 nM, and the final concentration of the CDs was 58 µg mL^−1^. After incubation, the bacteria were centrifuged (6000 rpm, 6 min) and washed three times with PBS containing 0.1% Triton X‐100 to avoid any adsorption of the Cy3‐ASO/CDs on the bacterial cells. The system was finally resuspended in PBS. The delivered amount of the Cy3‐ASO to *V. anguillarum* was then assessed using flow cytometry.

To optimize the concentration of the CDs for delivering the ASO, the fluorescent Cy3‐ASO (10 µM, 50 µL) was incubated with varying amounts of the CDs for 30 min. The *V. anguillarum* at a density of 10^6^ CFU mL^−1^ in LB liquid medium (900 µL) was cultured with the above mixture (100 µL) at 28 °C for 1 h. The final concentration of Cy3‐ASO was 500 nm, and the final concentration of the CDs was varied at 0, 14.5, 29, 58, 145, and 290 µg mL^−1^. After incubation with the Cy3‐ASO/CDs, the *V. anguillarum* suspension was centrifuged (6000 rpm, 6 min) and washed three times with PBS containing 0.1% Triton X‐100 to remove the Cy3‐ASO/CDs adsorbed on the bacteria, and finally resuspended in PBS. The amount of Cy3‐ASO delivered to *V. anguillarum* was assessed using flow cytometry.

### 2.7 Real‐Time Quantitative Reverse Transcription‐PCR (RT‐qPCR)

The ASO/CDs (100 µL, CDs: ASO = 20:1 (mass ratio)) were added to the *V. anguillarum* suspension (900 µL, 10^6^ CFU mL^−1^). In the system (1 mL), the concentrations of the ASO and the CDs were 500 nM and 58 µg mL^−1^, respectively. The mixture was incubated at 28 °C for 2 h. Parallelly, water (100 µL), the ASO (100 µL, 5 µM), the CDs (100 µL, 580 µg mL^−1^), and a random DNA sequence (c‐DNA) loaded with the CDs (c‐DNA/CDs) were separately incubated with the identical *V. anguillarum* suspensions at 28 °C for 2 h, serving as the controls. Subsequently, each system was centrifuged (6000 rpm, 6 min), washed with PBS containing 0.1% Triton X‐100, and finally resuspended in PBS (100 µL). The total RNA of the *V. anguillarum* was extracted using 1 mL of Trizol reagent. HiScript III All‐in‐one RT SuperMix Perfect for qPCR was then used to reversely transcribe the total RNA into cDNA. Finally, upstream and downstream primers were added, and amplification was performed using ChamQ SYBR Color qPCR Master Mix (Low ROX Premixed). The value of each cycle threshold was measured using qPCR. Using 16S rRNA as a reference gene, the relative expression of the target gene (*empA*) was quantified using the 2^−ΔΔCt^ method.^[^ ^28^ ^]^ For the gene silencing experiments using the CDs loading other ASOs (ASO_1_/CDs, ASO_2_/CDs, and ASO_3_/CDs), the identical procedure was applied.

### 2.8 Phenotype Assessment for the Gene‐Inactivated *V. Anguillarum*

The ASO/CDs (100 µL, with a CD‐to‐ASO mass ratio of 20:1) were added to a suspension of *V. anguillarum* (900 µL, 10^6^ CFU mL^−1^), resulting in final concentrations of 500 nM for ASO and 58 µg mL^−1^ for the CDs. This mixture was incubated at 28 °C for 2 h. The other groups of *V. anguillarum* were separately mixed with water (100 µL), the CDs (100 µL, 580 µg mL^−1^), the ASO (100 µL, 5 mM), and the c‐DNA/CDs (100 µL, c‐DNA: 500 nM, CDs: 570 µg mL^−1^) with a CD‐to‐c‐DNA mass ratio of 20:1, followed by incubation at 28 °C for 2 h. Following incubation, 20 µL of each bacterial suspension was plated onto LB agar containing skim milk (3%) and NaCl (2%) and then incubated for 24 h. Phenotypic changes in *V. anguillarum* were assessed by the appearance of a clear zone around the bacterial colonies.^[^ ^29^ ^]^

### 2.9 Synthesis of the Adhesive Hydrogel

The adhesive hydrogel used in this work was synthesized by following a reported method with slight modification.^[^ ^30^ ^]^ In brief, TA (0.3 g), chitosan (0.3 g), and aluminum nitrate nanohydrate (80.0 mg) were dissolved in water (10 mL) under stirring. Then, AA (3.0 g) and *N*,*N*‐methylenebisacrylamide (10.0 mg) were added under stirring (20 min). Subsequently, APS solution (34.8 mg mL^−1^, 2.3 mL) was added dropwise, followed by sonication to remove air bubbles. The resulting mixture was poured into a mold and heated in a water bath as the temperature was gradually increased from 60 to 80 °C. The system was eventually kept at 80 °C for 30 min before cooling down to room temperature.

### 2.10 Measurements of the Adhesive Properties of the Hydrogel

Two pieces of substrates were bonded together using hydrogel, with a bonding area of 15 × 15 mm^2^. A finger pressure of about 1 kPa was applied for 30 s at room temperature in air. Subsequently, the external pressure was removed. After 1 min, the adhering substrates were then subjected to shear stress measurements with the testing stand. The maximum tensile stress required to break the adhesive joints was measured at a rate of 100 mm min^−1^. The average adhesion performance was calculated based on at least three replicates.

To measure the adhesion strength of the hydrogel in seawater, the hydrogel‐adhering porcine skin was immersed in seawater for different times (0, 0.5, 1, 1.5, and 2 h) before shear stress measurements with the above procedure. To assess the adhesion strength of the hydrogel to porcine skin under simulated underwater conditions, varying flow dynamics were mimicked by adjusting the speeds (0, 500, 1000, and 1500 rpm min^−1^) of a magnetic stirrer. The hydrogel‐adhering porcine skin was placed in the simulated underwater environment before shear stress measurements.

### 2.11 Cultivation of Turbots

Juvenile turbots (≈25.0 g) purchased from the Lingyue Seafood Sales Center in the Rizhao Economic and Technological Development Zone were used as the animal model. All the juvenile turbots were temporarily reared in tanks for at least one week before the experiments and were fed once a day. The seawater used for culture was obtained from Qingdao offshore. The temperature of the seawater was maintained at 18 ± 2 °C, the dissolved oxygen was ≥4.0 mg L^−1^, the salt concentration was ≈32% and the daily water exchange was ≥80%. The experimental plan was approved by the Animal Care and Use Committee of the Experimental Animal Ethics Review Form of the College of Food Science and Engineering, Ocean University of China (approval number: SPXY2024052801). All the experiments were conducted in accordance with the Guide for the Care and Use of Laboratory Animals of the National Institutes of Health.

### 2.12 Determination of the Median Lethal Dose (*LD*_50_)

Uniform, healthy, and vigorous juvenile turbot were selected and randomly divided into seven groups, each involving 10 fishes. After anesthetizing the turbot with eugenol (100 mg L^−1^), sterilized scalpels were used to make incisions along the dorsal fin, ensuring consistent wound length and depth.^[^ ^31^ ^]^ The seven groups of the turbot were then placed in seawater containing different concentrations (0, 5×10^3^, 5×10^4^, 5×10^5^, 5×10^6^, 5×10^7^, and 5×10^8^ CFU mL^−1^) of *V. anguillarum* for 24 h. Afterward, all the turbots were transferred to seawater free of *V. anguillarum* for further rearing for 7 days, and the number of deaths in each group of turbots was recorded daily. The LD_50_ of *V. anguillarum* was calculated using the modified Karber method,^[^ ^32^ ^]^ which reads:

(1) $$LD_{50\;}=\mathrm{lo}g^{-1}\left[X_{m\;}-i\left(\sum p-\;0.5\right)\right]$$

where *X*_m_ represents the logarithmic value of the dose in the highest dose group, *i* indicates the difference between the logarithmic values of two neighboring dose groups, and ∑*p* is the sum of the mortality rates of turbot in all the groups.

### 2.13 Prevention of Fin Rot Disease in Turbot

Uniform, healthy, and vigorous juvenile turbot were selected and randomly divided into eight groups, each involving 10 fishes. After anesthetizing the turbots with eugenol (100 mg L^−1^), sterilized scalpels were used to make incisions along the dorsal fin, ensuring consistent wound length and depth. The first group of fish was uninfected; While the other seven groups were then placed in seawater containing *V. anguillarum* (LD_50_ = 2 × 10⁶ CFU mL^−1^) for 24 h to induce infection. Following the infection, the fish were transferred to seawater free of the *V. anguillarum* for further rearing. The fish wounds in one of the seven groups of infection were treated with the hydrogel‐sealed ASO/CDs (ASO: 500 nM, CDs: 58 µg mL^−1^, 50 µL) with a CD‐to‐ASO mass ratio of 20:1 for seven consecutive days with an interval of 12 h. Each treatment of the wound with the ASO/CDs was performed after wound cleaning with sterile water, followed by immediate hydrogel sealing. The other five groups were separately treated with sterile water (50 µL), the CDs (58 µg mL^−1^, 50 µL), the ASO (500 nM, 50 µL), the ASO/CDs (ASO: 500 nM, CDs: 58 µg mL^−1^, 50 µL) with a CD‐to‐ASO mass ratio of 20:1, and the hydrogel. The last group of fish was treated with oxolinic acid baths for 3 h daily at a concentration of 3 mg L^−1^ over a consecutive 7‐day period.^[^ ^33^ ^]^

### 2.14 Statistical Analysis

The results presented in this study were obtained from three independent experiments. The error bars represent the standard deviations observed in these independent experiments. Statistical analyses were conducted using Prism 8 (GraphPad Software Inc., San Diego, CA, USA) with a one‐way analysis of variance. The *P* value was used to determine statistical significance: ns, not significant; ^*^ *P* < 0.05, ^**^ *P* < 0.01, ^***^ *P* < 0.001.

## 3 Results and Discussion

### 3.1 Synthesis and Characterization of the CDs

The water‐soluble CDs were synthesized through a straightforward one‐step hydrothermal method utilizing CA and amino‐rich PEI as the precursors (Figure 2a).^[^ ^11^ ^]^ The statistical size analysis for the CDs imaged by TEM reveals an average diameter of 3.1 nm for the CDs, accompanied by a relatively narrow size distribution (Figure 2b). The XRD pattern shows a broad peak centered at 20° (2*θ*) (Figure S1, Supporting Information), corresponding to an interlayer spacing of 0.44 nm (Figure 2b), which is larger than the 0.33 nm spacing between (002) planes in bulk graphite.^[^ ^34^ ^]^ The increase in spacing is attributable to the incorporation of oxygenated and nitrogenated groups along the edges of the CDs.^[^ ^34^, ^35^ ^]^ Spectral characterizations reveal that the CDs exhibit absorption peaks at 351 and 235 nm (Figure S2, Supporting Information). The peak at 235 nm is likely attributed to the *π*–*π* ^*^ transition of C═C within the particle core.^[^ ^36^ ^]^ The XPS spectra indicate that the major constituent elements of the CDs are C (68.88%), N (16.06%), and O (15.06%) (Figure S3, Supporting Information). The FTIR spectra (Figure S4, Supporting Information) demonstrate that the CDs contain O─H/N─H (3450–3100 cm^−1^), ─CH_3_/─CH_2_─ (3000–2700 cm^−1^), C═O/C═N/C═C (1755–1670, 1690–1640, and 1680–1620 cm^−1^), C─N (1420–1350 cm^−1^), and O─C─O (1130–1060 cm^−1^) stretching vibrations. These FTIR peaks are consistent with those of previously reported CDs.^[^ ^11^, ^37^ ^]^ Additionally, the FTIR characterization explains the presence of the absorption peak at 351 nm, which can be attributed to the n‐π^*^ transition of C═O in the surface defects.^[^ ^38^ ^]^ According to the fluorescence measurements,^[^ ^39^ ^]^ the CDs exhibit excellent stability and durability at room temperature (Figure S5, Supporting Information).

### 3.2 Loading Capability of the ASO by the CDs

To develop an ASO‐delivery system for underwater wound treatment, fin rot disease in turbot caused by infection of *V. anguillarum* was employed as a model. Several ASO sequences were designed for the optimization of regulation of the *empA* gene in *V. anguillarum* (Tables S1, S2, Supporting Information). By taking advantage of gel (1% agarose) electrophoresis, we could evaluate the loading capacity of the CDs for ASO. As shown in Figure 2c, free ASO in lane 1 indicates a high fluorescence intensity band. The intensity of the ASO band in lanes 2–7 gradually decayed as the amount of the CDs increased. When the mass ratio of the CDs to the ASO reached 5:1 (lane 6) or higher (lane 7), no free ASO bands were observed, indicative of the complete loading of the ASO on the CDs and the formation of the ASO/CDs complexes. The zeta potentials of the ASO, CDs, and ASO/CDs were ‐5.2, 7.6, and 2.0 mV, respectively (Figure 2d). The positive charge of the ASO/CDs is thought to be advantageous in bacterial surface adsorption and subsequent internalization. In addition, the loading of the ASO has little effect on the emission of the CDs (Figure S6, Supporting Information). We also observed that the ASO/CDs exhibit excellent stability in seawater (Figures S7, S8, Supporting Information).

### 3.3 ASO/CDs Internalized into *V*. *anguillarum*

**Figure 3:**
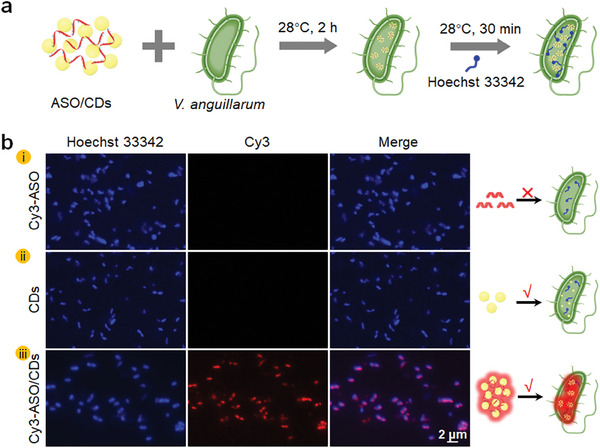
Internalization of the ASO/CDs into the *V. anguillarum*. a) Schematic illustration of the procedure for internalization of the ASO/CDs and Hoechst 33342 dye into the *V. anguillarum*. b) Fluorescence images of the *V. anguillarum* that are treated with the ASO (i), CDs (ii), and ASOs/CDs (iii) for 2 h and then stained with Hoechst 33342 dye for 30 min. Hoechst 33342 dye emits blue fluorescence under the excitation wavelength of 353 nm. Cy3‐ASO emits red fluorescence under the excitation wavelength of 550 nm. Colocalized areas of the blue Hoechst 33342 channel and red Cy3 channel are rendered in pink.

In addition to the tiny size, the CDs demonstrate the capability of enhancing the permeability of the cytoplasmic membranes of bacteria, where *V. anguillarum* was used as the model (Figure S9, Supporting Information). The results further inspired us to exploit the CDs as the carriers to deliver ASO into bacterial cells. Fluorescence microscopy was subsequently employed to perform colocalization analysis, assessing whether the ASO/CDs were internalized by the *V. anguillarum*. Upon incubation of the ASO/CDs with the bacteria for 2 h, Hoechst 33342 was used to stain live bacterial cells, emitting blue fluorescence (**Figure** **3**a).^[^ ^40^ ^]^ As can be seen in Figure 3b, red fluorescence from the ASO/CDs‐treated *V. anguillarum* was observed, attributable to the emission from the Cy3‐labeled ASO.^[^ ^41^ ^]^ However, no red fluorescence could be detected from the bacteria treated with either the CDs or the Cy3‐labeled ASO alone. The sharp contrast implies the necessity of the CDs in the internalization of the ASO into the bacteria. Indeed, PBS containing 0.1% Triton X‐100 was used to wash the ASO/CDs‐treated *V. anguillarum* before fluorescence observation to rule out the possibility of bacterial surface adsorption of the ASO/CDs.^[^ ^42^, ^43^ ^]^ As a consequence, the fluorescence emission in Figure 3b (iii) directly evidences the internalization of the ASO into the *V. anguillarum*.

### 3.4 Optimization of Internalization Conditions for the ASO/CDs by *V. anguillarum*

**Figure 4:**
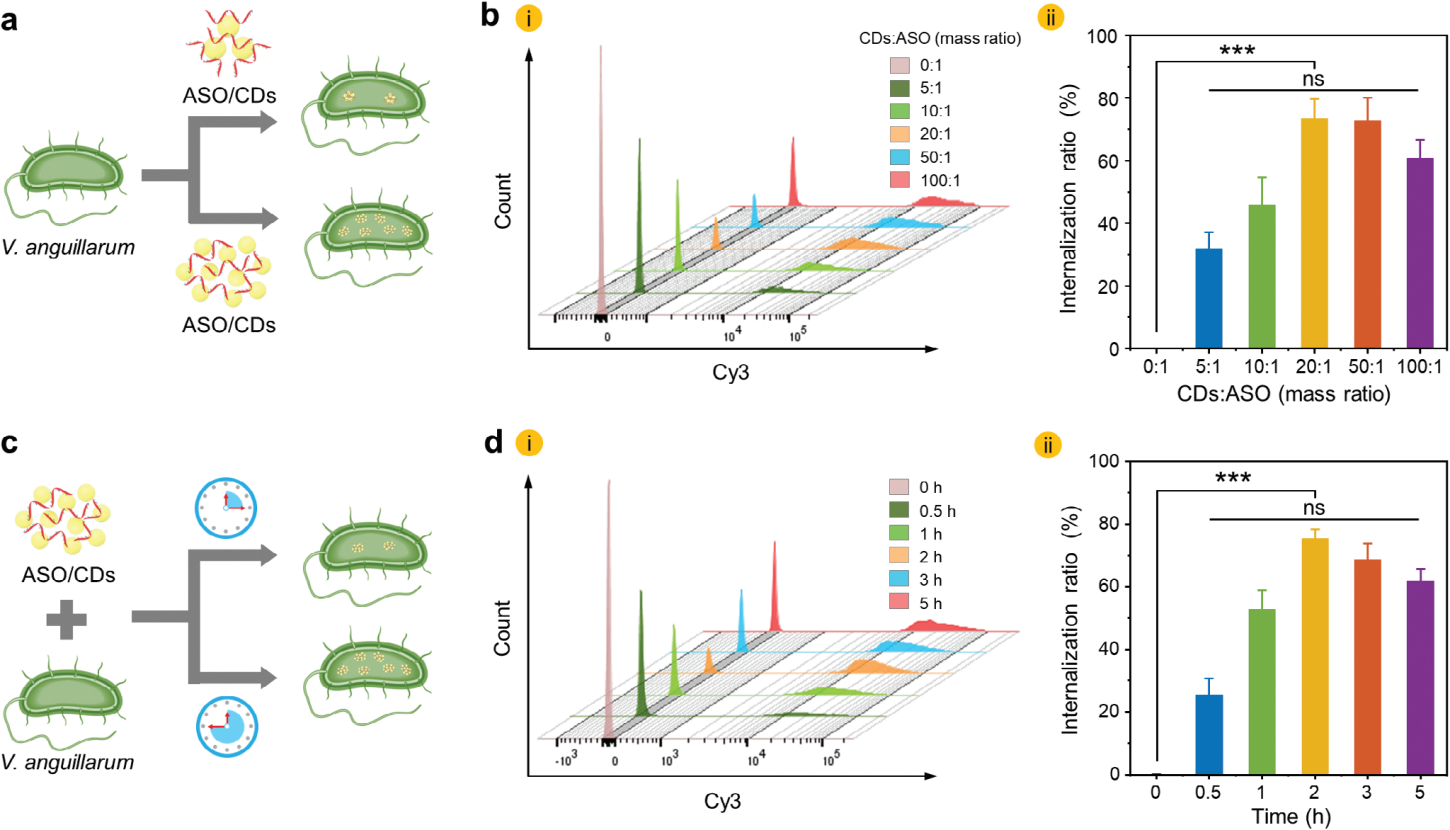
Optimization of the internalization of the ASO/CDs into the *V. anguillarum*. a) Schematic illustration of the procedure for internalization of the ASO loaded with the different amounts of the CDs into the *V. anguillarum*. b) Flow cytometry analysis of the internalization proportion of the *V. anguillarum* ASO/CDs at different CDs‐to‐ASO mass ratios (CDs: ASO = 0:1, 5:1, 10:1, 20:1, 50:1, 100:1) after incubation for 2 h. i) Representative flow cytometry histograms. ii) Histogram depicting the internalization rate of the *V. anguillarum* at different CDs‐to‐ASO mass ratios. c) Schematic illustration of the procedure for internalization of the ASO/CDs into the *V. anguillarum* at different incubation times. d) Flow cytometry analysis of the internalization proportion of the *V. anguillarum* at the CDs‐to‐ASO ratio of 20:1 after incubation for different times (0, 0.5, 1, 2, 3, and 5 h). (i) Representative flow cytometry histograms. (ii) Histogram of internalization rate of the *V. anguillarum* vs incubation time.

The amount of CD carriers plays a critical role in determining the delivery efficiency for the ASO into *V. anguillarum* (**Figure** **4**a). Flow cytometry measurements shown in Figure 4b‐i, reveal that the fluorescence intensity of the Cy3‐labeled ASO indicates a biphasic trend as the mass ratio of the CDs to the ASO increases from 0:1 to 100:1. Without the use of the CDs, the intensity of the fluorescence from the corresponding *V. anguillarum* is negligible, which implies that ASO by itself is hardly internalized into the *V. anguillarum* (Figure 4b‐i). As the amount of the CDs increases, the fluorescence intensity of the Cy3‐labeled ASO internalized into the bacteria increases monotonically until the mass ratio is as high as 20:1. The ratio of the *V. anguillarum* internalized with the ASO/CDs reaches 73.4%; While it tends to decay upon further increase in the amount of the CDs (Figure 4b‐ii), which may be ascribed to the inhibited bacterial activity with concentrated CDs and thereby depressed internalization (Figure S10, Supporting Information).

The efficiency of ASO/CDs‐internalization and bacterial growth are competing factors during the incubation. To this end, the effect of incubation time on the internalization efficiency of the *V. anguillarum* (Figure 4c). Interestingly, we also observed a biphasic trend in the fluorescence intensity of the Cy3‐labeled ASO as the incubation time increased (Figure 4d‐i). After incubation for 0.5 h, the ratio of the ASO/CDs‐internalized *V. anguillarum* is 25.4% (Figure 4d‐ii). The fluorescence intensity of the *V. anguillarum* becomes the strongest after 2 h of incubation, which is in line with the highest ratio (75.4%) of the ASO/CDs‐internalized *V. anguillarum*. With the further extension of incubation time, however, the internalization efficiency gradually decreases. The results can be ascribed to competition between the fixed amount of ASO/CDs and the fast‐growing number of *V. anguillarum* in the system. Indeed, it is revealed that the CDs at the test concentration (58 µg/mL, CDs: ASO = 20:1 in mass ratio) have a negligible effect on the growth of the *V. anguillarum* (Figure S10, Supporting Information).

### 3.5 Gene Silencing Effects of the ASO/CDs

**Figure 5:**
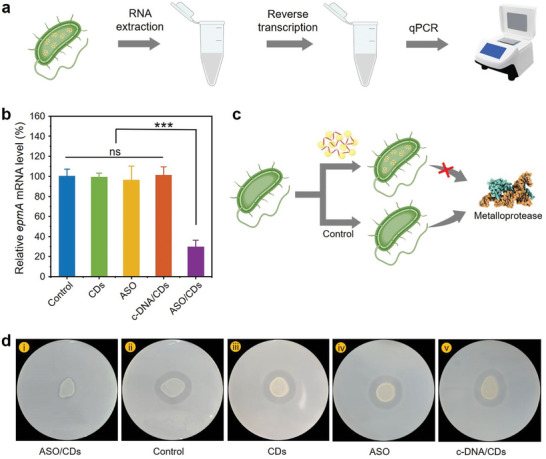
Application of the ASO/CDs to targeted gene inactivation of the *V. anguillarum*. a) Schematic illustration for the measurement of the transcription levels of the *epmA* gene in the ASO/CDs‐internalized *V. anguillarum* using RT‐qPCR. b) Histogram of the transcription levels of the *epmA* gene in the *V. anguillarum* upon treatment for 2 h with water (Control), the CDs, the ASO, the c‐DNA/CDs, and the ASO/CDs. c) Schematic illustration of the inhibition of metalloprotease production in V. anguillarum using the ASO/CDs. d) Digital photographs illustrating the phenotype change of the *V. anguillarum* upon treatment with the ASO/CDs (i), water (ii), the CDs (iii), the ASO (iv), and the c‐DNA/CDs (v). The area of the transparent zone surrounding *V. anguillarum* plated on LB agar containing skim milk (3%) and NaCl (2%) corresponds to the quantity of *epmA*‐coded metalloproteinases produced by *V. anguillarum*.

It has been recognized that the *empA* gene is responsible for the translation to extracellular metalloprotease which exhibits high proteolytic activity. It can cause degradation of fish mucus and thereby tissue damage, eventually leading to systemic infection and mortality in fish populations.^[^ ^44^ ^]^ We investigated the impact of the internalized ASO/CDs in *V. anguillarum* on the expression of *empA* using RT‐qPCR (**Figure** **5**a). The silencing ASO sequence for the *empA* gene was optimized for the subsequent experiments (Figure S11, Supporting Information). As shown in Figure 5b, the *V. anguillarum* co‐incubated with free ASO and the ASO/CDs indicate a decrease in the transcript level of *empA* by 4.7% and 71.2%, respectively. The efficient silencing by the ASO/CDs suggests the significance of the CD carriers in delivering the ASO to the *V. anguillarum* and the subsequent gene regulation. Noteworthy, it is also discovered that the gene expression of *empA* in the *V. anguillarum* treated with the c‐DNA/CDs remains constant compared with the control group, directly evidencing the targeted gene silencing in the bacteria by the ASO/CDs (Figure 5c). In a phenotype test for *V. anguillarum*, additionally, we observed that the transparent zone surrounding *V. anguillarum* treated with the ASO/CDs was significantly smaller than that of treated with water, the CDs, the ASO, and the c‐DNA/CDs (Figures 5d). The results further indicate that the ASO/CDs effectively inhibit the production of extracellular metalloproteinases coded by the *empA* gene in *V. anguillarum*, agreeing well with the RT‐qPCR measurements.

Inspired by the efficient gene inactivation, we became interested in the mechanism of gene inactivation in the bacteria by the ASO/CDs. To examine the dependence of the ASO's activity in gene regulation on the CDs, we tested whether the ASO can be released from the CDs inside the bacteria. The Cy3‐ASO/CDs were subjected to a reaction with the *V. anguillarum* lysates (Figure S12a, Supporting Information). The significant fluorescence enhancement in the supernatant indicates the release of the ASO from the CDs upon delivery into *V. anguillarum* (Figure S12b, Supporting Information). This result rationalizes the subsequent pairing of the ASO with the target mRNA inside the bacteria after being carried into the bacteria by the CDs, which eventually causes inhibition of the translation of the mRNA into pathogenic protein through steric hindrance and/or RNase H‐mediated mRNA cleavage.^[^ ^14^ ^]^

**Figure 6:**
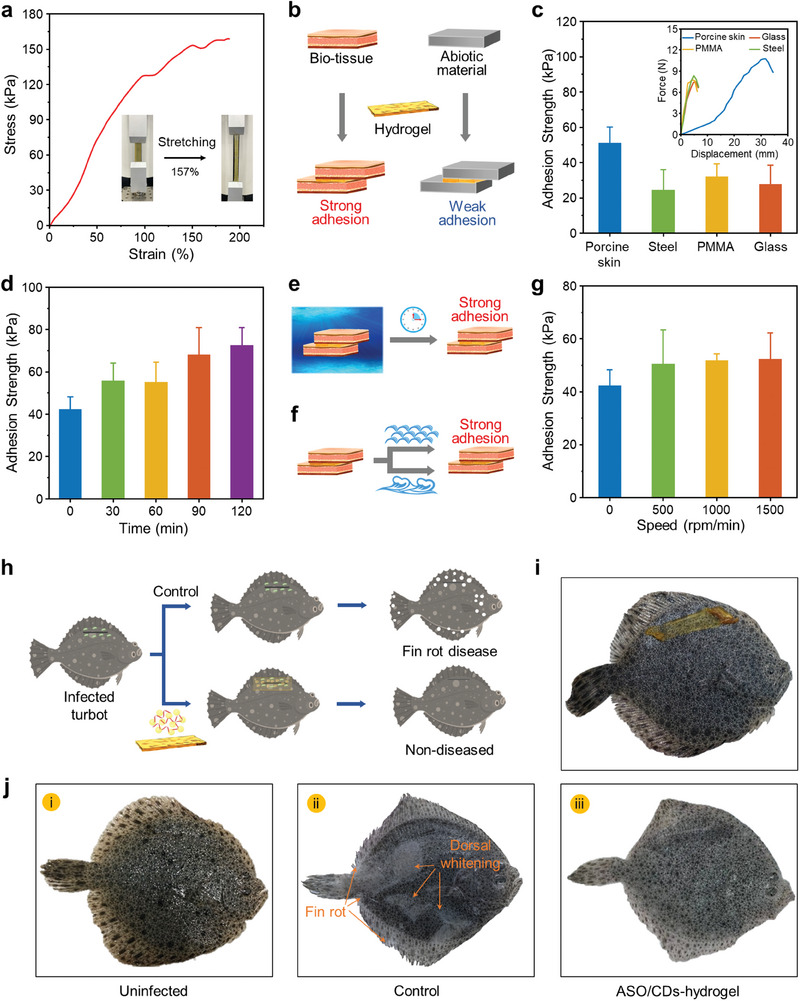
Characterizations of the hydrogel and the application of the hydrogel‐sealed ASO/CDs for turbot fin rot disease prevention. a) Stress‐strain curves of the hydrogel upon tensile loading at a fixed strain rate of 5 mm min^−1^. The inset shows the high stretchability of the hydrogel. b) Schematic illustration of the adhesion properties of the hydrogel to different substrates. c) Histogram depicting the adhesion strength of the hydrogel to porcine skin, steel, PMMA, and glass. d) Histogram depicting the adhesion strength of the hydrogel to porcine skin after immersion in seawater for different times (0, 0.5, 1, 1.5, and 2 h). e) Schematic illustration of the adhesion of the hydrogel to porcine skin after immersion in seawater for a given time. f) Schematic illustration of the adhesion of the hydrogel to porcine skin in seawater under different flow dynamics. g) Histogram depicting the adhesion strength of the hydrogel to porcine skin in seawater under different flow dynamics mimicked by adjusting the speeds (0, 500, 1000, and 1500 rpm) of a magnetic stirrer. h) Schematic illustration of the prevention of turbot fin rot disease infected by the *V. anguillarum* using adhesive hydrogel‐sealed ASO/CDs. i) A typical image of the adhesive hydrogel adhering to the ASO/CDs‐treated wound site of the *V. anguillarum*‐infected turbot. j) Representative photographs of the scratched turbot: uninfected (i), infected and treated with water (Control, ii), and infected and treated with the composite (iii), which were captured five days after scratching.

To apply the ASO/CDs for targeted inhibition of *V. anguillarum* at wound sites underwater, a hydrogel bandage with excellent mechanical properties and underwater adhesiveness was prepared to maintain the concentration of ASO/CDs at wound sites for efficient wound healing. The hydrogel has a tensile strength of up to 188.9 kPa, an elongation of ≈157% (**Figure** **6**a), and a maximum stress of 2000 kPa under 50% compressive deformation (Figure S13, Supporting Information). The adhesion of the hydrogel to different substrates was then determined (Figure 6b). Importantly, the hydrogel exhibits a much stronger adhesion to the bio‐tissue (porcine skin) than abiotic materials including steel, polymethyl methacrylate (PMMA), and glass, with a maximum strength of over 50 kPa, (Figure 6c). This is because the hydrogel can adhere to porcine skin through a combination of hydrogen bonding, electrostatic interactions, and dynamic covalent bonding, whereas its adhesion to abiotic materials (e.g., glass) relies solely on hydrogen bonding.^[^ ^30^ ^]^ Interestingly, we observed that the hydrogel's adhesion to porcine skin in seawater becomes stronger with time, reaching 72.4 kPa after incubation in seawater for 2 h (Figure 6d,e). The results can be ascribed to the water diffusion‐driven interfacial bonding between the hydrogel and porcine skin during the solvent exchange process, resulting in enhanced adhesion.^[^ ^45^ ^]^ The adhesion of the hydrogel to porcine skin in seawater under different flow dynamics was also tested (Figure 6f and Video S1, Supporting Information). Negligible changes in the adhesion strength were obtained (Figure 6g). Also note that the hydrogel exhibits a low swelling ratio when placed in seawater, which is only 3.4% after 2 h (Figure S14, Supporting Information).

We subsequently evaluated the efficacy of the ASO/CDs in the prevention of fin rot disease in cultured turbots (Figure 6h). Caused by infection of *V. anguillarum*, fin rot is highly prevalent in turbot aquaculture and can result in high mortality rates and substantial economic losses.^[^ ^46^ ^]^ The model of fin rot disease in turbot was established by *V. anguillarum*‐infection of the scratch along the dorsal fin. A group of the turbots were treated with the ASO/CDs, and the adhesive hydrogel was employed for sustained retention of the locally concentrated ASO/CDs at the wound site of the *V. anguillarum*‐infected turbot (Figure 6i and Videos S2, S3, Supporting Information). The hydrogel exhibits excellent adhesive properties to turbot (Video S4, Supporting Information). Within 2 h, a retention rate of 98.3% was determined for the hydrogel (Figure S15, Supporting Information). Numerous treatments for the scratched turbots were conducted, and morbidity and mortality among the groups were surveyed (Table S3, Supporting Information).

Without the *V. anguillarum* infection, the turbots show strong vitality, with no signs of fin rot observed after five days (Figure 6j‐i; Figure S16a, Supporting Information). In contrast, turbots with wounds infected by *V. anguillarum* in the control group (treated with water) exhibit symptoms such as dorsal whitening, fin rot, and, in some cases, death (Figure 6j‐ii; Figure S16b, Supporting Information). Interestingly, no fin rot is observed in turbot treated with the ASO/CDs‐hydrogel composite (Figure 6j‐iii; Figure S16c, Supporting Information), despite a slight decline in vitality. No death is observed in this group, resembling the group treated with oxolinic acid (Figures S16d, Supporting Information). It is noteworthy that turbots treated with the CDs, the ASO, the hydrogel, or the ASO/CDs alone show outcomes similar to those of the control group (Figure S16e–h, Supporting Information), underscoring the essential role of the ASO/CDs‐hydrogel composite. We also increased the concentration of the ASO/CDs by five‐ and tenfold to evaluate the preventive effect on turbots. Still, no effectiveness against fin rot disease was observed (Figures S17, Supporting Information). The results highlight the critical role of the hydrogel in preserving the effective concentration of the ASO/CDs, thereby enhancing their performance.

Indeed, our results also indicate that both the CDs and the hydrogel afford excellent biocompatibility. Considerably high cell viabilities after their incubation for 24 h with CSGC and HepG2 cells were obtained (Figure S18,b, Supporting Information), consistent with the low cytotoxicity of the ASO/CDs‐hydrogel composite (Figure S18c, Supporting Information). The treatment of a turbot with the ASO/CDs‐hydrogel composite for seven consecutive days costs ≈1.1 RMB only. It is thereby deduced that the use of the ASO/CDs sealed with the hydrogel, as a non‐antibiotic therapeutic intervention, effectively prevented the onset of fin rot in the *V. anguillarum*‐infected turbot, demonstrating its potential as a safe and cost‐effective alternative to traditional antimicrobial approaches. The absence of death in the treated group also underscores the safety and efficacy of the ASO/CDs in mitigating infection‐related damage.

## 4 Conclusions

Current control strategies for underwater treatment of bacterial diseases, primarily reliant on antibiotics, face significant challenges such as poor efficacy, environmental pollution, and high costs. By employing fin rot disease in turbot caused by infection of *V. anguillarum* as a model, this study presents a conceptually new approach utilizing nanoparticle‐based delivery of specific ASO for precise and efficient (~71.2%) gene silencing of pathogenic bacteria, assisted with an adhesive hydrogel with the localization capability in a submerged environment. The dual‐action system overcomes several key challenges associated with underwater treatment, providing three key advantages in the field. First, by developing the CD‐based nucleic acid delivery systems for bacterial cells, we have achieved a breakthrough by transforming bacterial gene silencing—previously restricted to laboratory settings—into a versatile and scalable solution for real‐world disease prevention. While demonstrated with a single pathogen gene, the approach should hold potential for adaption to target multiple genes or pathogens within complex microbial communities. Second, the use of adhesive hydrogel ensures that the ASO/CDs are retained at high local concentrations at the infected wound site in turbots, providing prolonged action and targeted therapeutic outcomes. Adhesive hydrogels could also maintain high concentrations of bioactive agents at specific sites underwater for a range of biomedical, agricultural, and environmental applications, such as enhancing crop and coral resistance to pathogens, and their nutrient uptake as well. Third, the CD and hydrogel materials exhibit minimal ecological impact while improving significantly the curing efficiency for fin rot disease, underscoring their promise for reducing environmental contamination in various underwater therapeutic contexts. By advancing the delivery of gene‐targeting therapies in submerged conditions, this study opens new avenues for the treatment of bacterial infections in both aquatic ecosystems and human healthcare, offering a platform for future innovations in precision underwater medicine. It is also our hope that innovation and application of materials will inspire more interdisciplinary research endeavors on transformative advancement in the management of both human and aquatic organism diseases that align with global efforts to foster resilient ecosystems.

## Conflict of Interest

The authors declare no conflict of interest.

## Author Contributions

G.W. conceived and designed the research. G.W., G.X., and H.L. designed the methodology. Q.Z., M.L., and R.O. conducted the experiments. G.W, G.X., X.X., and W.Z. analyzed the data. G.W. and Q.Z. wrote the manuscript. All authors discussed the results and commented on the manuscript.

## Supporting information



Supporting Information

Supplemental Video 1

Supplemental Video 2

Supplemental Video 3

Supplemental Video 4

## Data Availability

The data that support the findings of this study are available from the corresponding author upon reasonable request.
