# Supplementary material for: Nanodot‐Inspired Precise Bacterial Gene Suppression in a Smart Hydrogel Bandage for Underwater Wound Healing
Source: Adv Sci (Weinh). 2025 Feb 14;12(13):2415169. doi: 10.1002/advs.202415169 (PMC11967816; doi:10.1002/advs.202415169)
Supplement: Supplementary file 1 — Supporting Information [file ADVS-12-2415169-s005.docx]

Nanodot-Inspired Precise Bacterial Gene Suppression in a Smart Hydrogel Bandage for Underwater Wound Healing

Qingsong Zhang,^1,2,3^ Menghan Lu,^1,2,3^ Richang Ou,^4^ Hong Lin,^3^ Guanhua Xuan,*^,3^ Xiudan Wang,^3^  Xiaofeng Xu,^4^ Weiwei Zhang,^5^ Guoqing Wang,*^,^^1,2,3^

^1^*MOE Key Laboratory of Evolution and Marine Biodiversity and Institute of Evolution and Marine Biodiversity, Ocean University of China, 5 Yushan Road, Qingdao 266003, China*

^2^*Laboratory for Marine Drugs and Bioproducts, Qingdao Marine Science and Technology Center, Qingdao 266237, China*

^3^*State Key Laboratory of Marine Food Processing & Safety Control, College of Food Science and Engineering, Ocean University of China, 1299 Sansha Road, Qingdao 266400, China*

^4^*College of Materials Science and Engineering, Ocean University of China, Qingdao 266100, China*

^5^*School of Marine Sciences, Ningbo University, 169 Qixingnan Road, Ningbo 315832, China*

Email: xuan[guanhua@ouc.edu.c](mailto:guanhua@163.com)n; gqwang@ouc.edu.cn

**Table of Contents**

Evaluation of the stability of the CDs…………………………………….…………………..S4

Assessment of the effect of the CDs on bacterial growth ……………..…………….…….….S4

Assessment of the effect of the CDs on membrane permeability of bacterial….….…………S4

Evaluation of the stability of the ASO/CDs in seawater………………………………………S5

Evaluation of ASO release from the CDs………………………….………………………….S5

Measurements of the mechanical properties of the hydrogel…………………………………S6

Measurements of the swelling ratio of the hydrogel………………………………………….S6

Measurements of the retention rate of the CDs sealed by the hydrogels …………………….S7

Evaluation of the concentrated ASO/CDs in preventing fin rot disease in turbot……………S7

Evaluation of the cytotoxicity…..………….……………………………..…………………..S8

Calculation of Molar concentration ratio of ASO to the CD……………….………………….S9

**Table S1** Sequence information of the nucleic acids used in this study.……….…….………S12

**Figure S1** The XRD spectrum of the CDs…………...……………………………………..S13

**Figure S2** The absorption spectrum of the CDs..……………………………….......…….…S14

**Figure S3** The XPS spectrum of the CDs..………….…………………….…..…………......S15

**Figure S4** The FTIR spectrum of the CDs...…………………….….………. ……………....S16

**Figure S5** Effects of temperature and storage time on the emission of the CDs………….….S17

**Figure S6** The emission spectra of the CDs and the ASO/CDs………………………...……S18

**Figure S7** The emission of the ASO/CDs in seawater with time………………………...…..S19

**Figure S8** The emission of the ASO/CDs in seawater at different salinities………………....S20

**Table S2** Sequence information of the *empA* gene in the *V. anguillarum*…………….…….S21

**Figure S9** The bacterial membrane permeability upon treatment with the CDs ……...……..S22

**Figure S10** The bacterial growth activity upon treatment with the CDs.…………….……..S23

**Figure S11** The gene silencing effect of different ASO sequences ………………...………S24

**Figure S12** Test of the release of the ASO from the CDs……………..……………………S25

**Figure S13** The stress-strain curve of the hydrogel at 50% compression……………..……S26

**Figure S14** The swelling ratio of the hydrogel in seawater………………………..………..S27

**Figure S15** The retention rate of the CDs sealed by the hydrogels……………….……..…..S28

**Table S3** The morbidity and mortality of the grouped turbots……………………………….S29

**Figure S16** Photographs of the scratched turbots captured upon different treatments……...S30

**Figure S17** Photographs of the scratched turbots treated with concentrated ASO/CDs……..S32

**Figure S18** Evaluation of the cytotoxicity ….………..…..……..…….……………………..S33

***Evaluation of the stability of the CDs*** The CDs were sealed and stored under different temperatures (20℃ and 37℃) and environmental conditions (light and dark). The fluorescence spectra of the CDs solution (100 μg/mL) were measured every 15 days. The stability of the CDs was evaluated based on changes in fluorescence intensity.[**Ref. 1, 2**]

**Ref. 1**: C. Wang, Z. Xu, H. Cheng, H. Lin, M. G. Humphrey, C. Zhang, A hydrothermal route to water-stable luminescent carbon dots as nanosensors for pH and temperature, *Carbon* **2015,** *82*, 87-95.

**Ref. 2**: S. Dua, P. Kumar, B. Pani, A. Kaur, M. Khanna, G. Bhatt, Stability of carbon quantum dots: a critical review, *RSC Adv.* **2023,** *13*, 13845-13861.

***Assessment of the effect of CD concentration on bacterial growth*** A log-phase *V. anguillarum* suspension (100 μL, 10^6^ CFU/mL) was separately mixed with the CDs solutions (100 μL) at different concentrations (0, 14.5, 29, 58, 1445, and 290 μg/mL) in a 96-well plate. The mixture was then incubated in a shaking incubator (28°C, 180 rpm). The absorbance of each mixture at 600 nm was measured at 1 h intervals over 5 h.

***Assessment of the Effect of the CDs on membrane permeability of bacteria*** When the cytoplasmic membrane is disrupted, *β*-galactosidase is released from the cell and catalyzes the hydrolysis of ONPG to produce the yellow o-nitrophenol.[**Ref. 3**] Therefore, the permeability of the cytoplasmic membrane was assessed by measuring *β*-galactosidase activity in *V. anguillarum* using ONPG, a substrate for cytoplasmic *β-*galactosidase. Briefly, *V. anguillarum* was cultured in LB medium containing 2% lactose and 2% sodium chloride until it reached the logarithmic growth phase. Then, the *V. anguillarum* suspension was centrifuged (6000 rpm, 6 min), and the strain was washed and resuspended in PBS (5 mM) containing 2 mM ONPG and 20 mM glucose. Next, 100 μL of the bacterial suspension (10^6^ CFU/mL) was parallelly mixed with the CDs (100 μL) to achieve a predetermined final concentration (0, 14.5, 29, 58, 145, and 290 μg/mL) in a 96-well plate. The absorbance of each mixture at 420 nm was measured at 5 min intervals over 60 min.

**Ref. 3**: H. J. Jian, R. S. Wu, T. Y. Lin, Y. J. Li, H. J. Lin, S. G. Harroun, J. Y. Lai, C. C. Huang, Super-cationic carbon quantum dots synthesized from spermidine as an eye drop formulation for topical treatment of bacterial keratitis, *ACS Nano* **2017,** *11*, 6703-6716.

***Evaluation of the stability of the ASO/CDs in seawater*** The ASO/CDs solution (ASO: 500 nM, CDs: 58 μg/mL) with a CD-to-ASO mass ratio of 20:1 was prepared using seawater with a salinity of 30‰, and the fluorescence intensity was measured after different incubation times (0, 0.5, 1, 1.5, and 2 h).

To test the stability of the ASO/CDs again salinity, the ASO/CDs solution (ASO: 500 nM, CDs: 58 μg/mL) with a CD-to-ASO mass ratio of 20:1 was also prepared using seawater with different salinities (0, 16, 32, 38, and 40‰). Indeed, it is recognized that the salinity of the global oceans typically ranges from 33‰ to 37‰.[**Ref. 4**]

**Ref. 4**: Sea water, *National Oceanic and Atmospheric Administration* **2023**. <https://www.noaa.gov/jetstream/ocean/sea-water>

***Evaluation of ASO release from the CDs*** The lysate of *V. anguillarum* was prepared using a protocol reported elsewhere. [**Ref. 5**] Initially, the suspension of a log-phase *V. anguillarum* (OD_600_ = 0.6, 1.0 mL) was centrifuged (8000 rpm, 5 min), followed by resuspension of the resultant pellet in lysis buffer (1.0 mL). The suspension was then placed on ice for 30 min. Disruption of the bacterial cells was performed using an ultrasonic homogenizer at 300 W in an ice bath, with 2 s pulses for a total of 80 cycles, with 15 s intervals between each pulse. The supernatant as the lysate of *V. anguillarum* was obtained by centrifugation (12000 rpm, 30 min, 4°C).

Cy3-ASO was incubated with the CDs in the dark for 30 min, in which the final concentrations of Cy3-ASO and the CDs were 20 ng/μL and 100 ng/μL, respectively. The system (100 μL) was then centrifuged (12000 rpm, 5 min) to remove the supernatant containing free Cy3-ASO. Subsequently, the lysate of *V. anguillarum* (100 μL) was mixed with the pellet (Cy3-ASO/CDs). The suspension was incubated at 28°C in the dark for 1 h, followed by centrifugation (12000 rpm, 5 min). The fluorescence of the supernatant containing released Cy3-ASO was measured. The pellet (Cy3-ASO/CDs) incubated with water (100 μL) was used as the control.

**Ref. 5**: Y. Yang, W. Li, Y. Li, W. Shi, J. Zhang, W. Dang, W. Zhang, Exogenous c-di-GMP inhibited the biofilm formation of Vibrio splendidus, *Microb. Pathog.* **2023**, *175*, 105981.

***Measurements of the*** ***mechanical properties of the*** ***hydrogel*** The tensile and compression properties of the hydrogel were characterized using the motorized testing stand, at a speed of 5 mm/min. The specification of the tested hydrogel sample was 80×15×1 (mm^3^) in the tests of the hydrogel’s tensile properties. For tests of compression properties, the size of the tested hydrogel sample was 15×15×6 (mm^3^). The properties of hydrogel producing 50% compressive strain was tested in tensile property tests. The mechanical properties of the hydrogel were calculated based on at least three replicates.

***Measurements of the swelling ratio of the hydrogel*** Fresh hydrogel samples with similar sizes in cubic shape were weighed (*W*_0_). They were immersed in seawater for different times (0.5, 1, 1.5, and 2 h). Each of the resulting weight (*W*_t_) was weighed after removing surface water. The swelling ratio of the hydrogel was then calculated as follows:

*Swelling ratio* (%) = (*W*_t_ ‒ *W*_0_)/*W*_0_ ×100

where *W*_0_ is the weight of the fresh hydrogel; *W*_t_ is the weight of the hydrogel after immersion in seawater.

***Measurements of the retention rate of the CDs sealed in the hydrogels*** Three uniformly healthy and vigorous juvenile turbots were randomly selected and anesthetized with eugenol (100 mg/L). Incisions of consistent length and depth were made along the dorsal fin using sterilized scalpels, and the fish were then placed into 1 L of seawater. The fish wounds were treated with the CDs solution (58 mg/mL, 50 μL) and sealed with the hydrogel. To analyze the leaked CDs, seawater samples were collected every 0.5 h for fluorescence measurements. The retention rate of the CDs sealed by the hydrogel was calculated based on the standard curve of the CDs solution.

***Evaluation of the concentrated ASO/CDs in preventing fin rot disease in turbot***

To validate the importance of the hydrogel in preserving the ASO/CDs at the turbot wound site, highly concentrated ASO/CDs were introduced to the *V. anguillarum*-infected turbots. In specific, the wounds of two groups of the turbots were treated with different concentrations (ASO: 2.5 mM, CDs: 290 μg/mL; ASO: 5 mM, CDs: 580 μg/mL) of the ASO/CDs with a CD-to-ASO mass ratio of 20:1 (50 μL) for seven consecutive days, with an interval of 12 h between treatments. Each treatment of the wound with the ASO/CDs was performed after wound cleaning with sterile water. Note that the concentrations of the ASO/CDs adopted are higher than that of the ASO/CDs sealed with the hydrogel by five and ten times.

***Evaluation of the cytotoxicity*** The cytotoxicity of the CDs, the hydrogel, and the ASO/CDs-hydrogel composite was evaluated using the CCK-8 assay kit in half-smooth tongue sole testicular cells (CSGC) and human hepatocellular carcinoma cells (HepG2). Briefly, CSGC cells were cultured in Leibovitz's L-15 medium supplemented with 10% FBS, 1% P/S, and 1% non-essential amino acids until they reached the logarithmic growth phase. Cell suspensions (100 μL, 5×10^4^ cells/mL) were dispensed into individual wells of a 96-well plate and incubated at 24°C for 24 h. The cells were washed with PBS. Subsequently, the CDs solution (100 μL) at final concentrations of 0, 60, 120, and 240 μg/mL was added to each well and incubated for different durations (12 and 24 h) in the incubator. Finally, CCK-8 solution was added to each well to achieve a final volume ratio of 10%, and incubated for 1h. Absorbance at 450 nm was measured using a microplate reader. Cell viability was calculated as follows:

*Cell viability* (%) = [*A*_1_ ‒ *A*_(control)_] / [*A*_0_ ‒ *A*_(control)_] × 100

where *A*_0_ is the absorbance of the mixture of the cells and CCK-8 solution, *A*_1_ is the absorbance of the mixture of the cells, CCK-8 solution, and the CDs, and *A*_(control)_ is the absorbance of the mixture of the culture medium and CCK-8 solution.

In the cytotoxicity test for the hydrogel, the identical procedure was applied, except for the varied final concentration (0, 100, 200, and 400 μg/mL) of the hydrogel used for incubation with CSGC. For the ASO/CDs-hydrogel composite, the same procedure was followed, except for the addition of the ASO/CDs (ASO: 500 nM, CDs: 58 μg/mL) and the hydrogel (200 μg/mL) for incubation with CSGC at different durations (0, 2, 4, and 8 h). Notably, hydrogels of different concentrations were prepared by multiple washings with ultrapure water, followed by lyophilization, and subsequent immersion in the culture medium for 72 h to produce a homogeneous suspension by stirring.

In the cytotoxicity assessments for the CDs, the hydrogel, and the ASO/CDs-hydrogel composite on HepG2 cells, the same procedures were adopted, except for a different culture medium (DMEM containing 10% FBS and 1% P/S) and a different temperature (37 °C).

***Calculation of Molar concentration ratio of ASO to the CD***

The CDs are primarily composed of C, N, and O, with approximate elemental ratios of 69:16:15 (**Figure S2**). Assuming that the CDs are composed of the @, the radius of the @ (*r_@_*) is calculated according to the following formula:

*r_@_* = (69×*r*_C_ + 16×*r*_N_ + 15×*r*_O_)/100 = 0.0677 nm

where *r*_C_ is the radius of C (~0.070 nm), *r*_N_ is the radius of N (~0.065 nm), *r*_O_ is the radius of O (~0.060 nm).

The volume of the @ (*V_@_*) is calculated according to the following formula:

$V_{@}=\frac{4}{3}\pi{r_{@}}^{3}$ = 0.0013 nm^3^

The relative atomic mass of the @ (*A_@_*) can be estimated according to the following formula:

*A_@_* = (69×*A*_C_ + 16×*A*_N_ + 15×*A*_O_)/100 = 12.92

where *A*_C_, *A*_N_ and *A*_O_ are the relative atomic masses of C (12), N (14), and O (16), respectively.

We assume that the CD is of spherical shape, and is composed of many @ in close proximity to each other. Since the diameter (*d*) of a single CD is characterized to be 3.1 nm (**Figure 2b**), the volume of a single CD (*V*) can be calculated by the following formula:

$V=\frac{4}{3}\pi({\frac{d}{2})}^{3}$ = 15.59 nm^3^

The relative mass of a single CD (*M_CD_*) can be calculated by the following formula:

*M_CD_* = (*V*/*V_@_*)×*A_@_* = 154940.62

The optimal mass ratio for the CDs loaded ASO is *m_CD_*:*m_ASO_* = 5:1 (**Figure 2c**). The relative molecular mass of a single ASO (*M_ASO_*) is 5805.7. Therefore, the molar concentration ratio of the CD to ASO (*C*_CD_:*C*_ASO_) can be calculated by the following formula:

*C*_CD_:*C*_ASO_ = (*m_CD_*/*M_CD_*) : (*m_ASO_/M_ASO_*) = 1:5.3

**Table S1** Nucleic acid sequences relevant to this study.

| Nucleic acid | Base sequence (5'→3') | Base number |
| --- | --- | --- |
| ASO | T*T*A*C*A*A*G*G*T*A*C*G*C*G*A*A*G*C | 18 nt |
| ASO_1_ | C*T*T*C*A*C*C*A*C*A*A*G*T*T*G*A*A*C | 18 nt |
| ASO_2_ | C*C*A*A*A*A*T*A*T*G*T*C*T*G*G*T*G*G | 18 nt |
| ASO_3_ | T*G*A*A*C*A*G*T*G*C*A*C*A*A*T*T*T*C | 18 nt |
| Control DNA oligonucleotide (c-DNA) | T*C*A*C*C*T*G*T*T*C*A*C*T*G*A*T*A*C | 18 nt |
| *empA* (forward) | GCAAAAGCGGCACAAGAC | 18 nt |
| *empA* (reverse) | GCATCACCTGAGCCCAAAC | 19 nt |
| 16S rRNA(forward) | GGAGTACGGTCGCAAGATTA | 20 nt |
| 16S rRNA (reverse) | TCGCTGGCAAACAAGGAT | 18 nt |

Note: The asterisk (*) represents the replacement of the non-bridging oxygen atom in the ASO phosphodiester bond with a sulfur atom. Note that ASO, ASO_1_, ASO_2_, and ASO_3_ were designed from the 1569^th,^ 337^th^, 1082^nd^, and 1739^th^ base positions of the *empA* gene sequence, respectively.


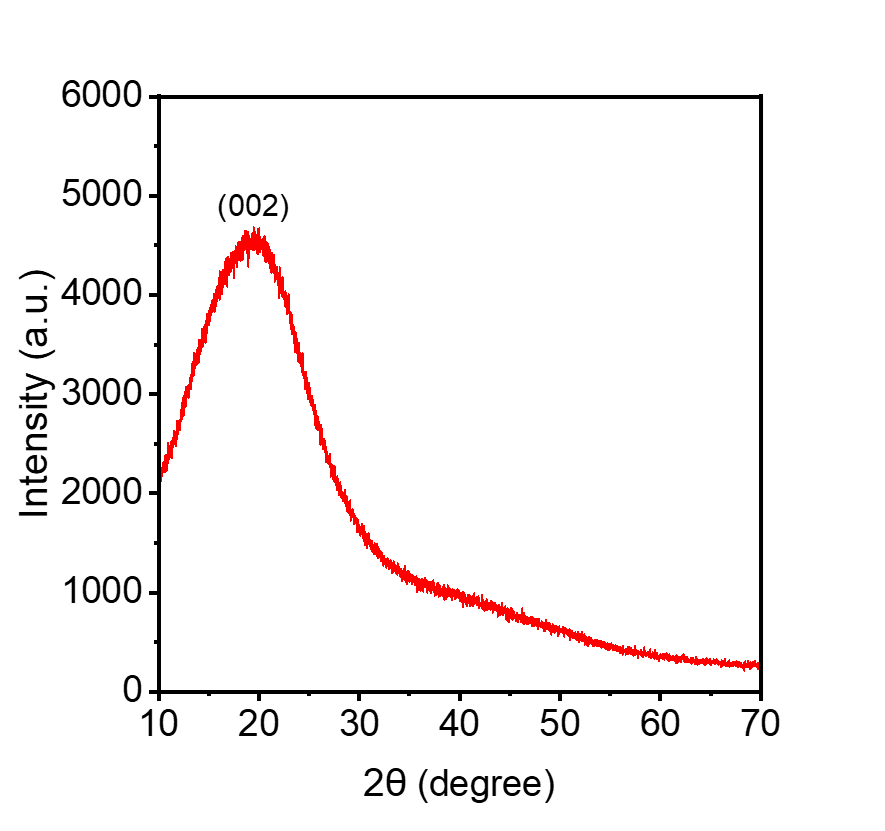


**Figure S1** The XRD spectrum of the CDs. The XRD pattern reveals a broad band centered at 20° (2*θ*).


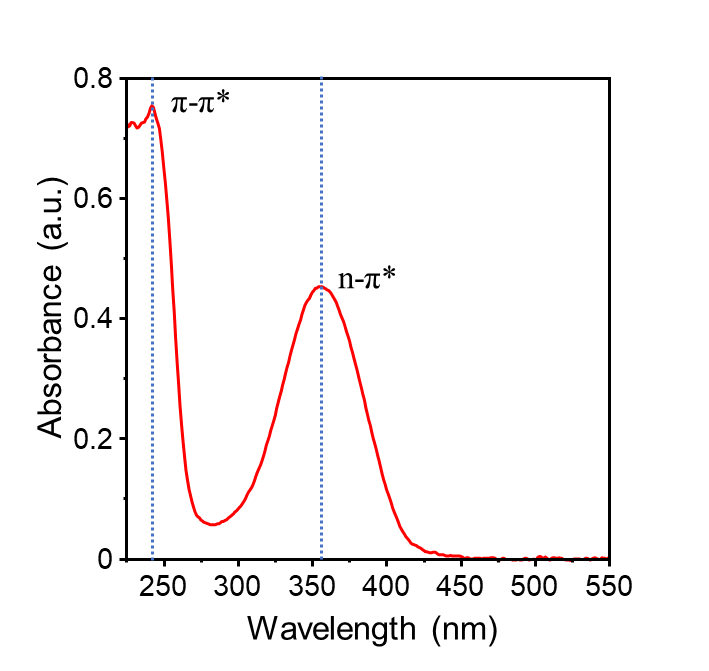


**Figure S2** The absorption spectrum of the CDs. The absorption spectrum shows that the CDs display absorption peaks at 351 nm and 235 nm.


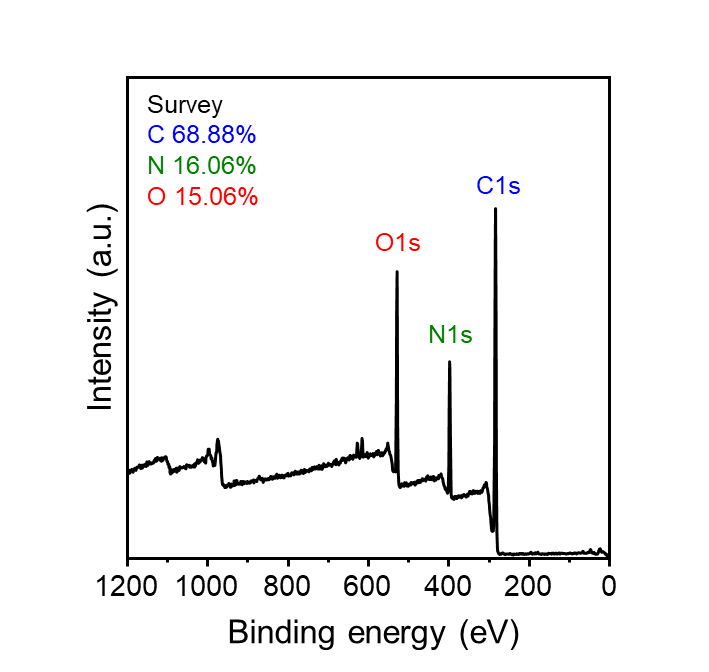


**Figure S3** The XPS spectrum of the CDs. The XPS spectrum reveals that the primary elements composing the CDs are C (68.88%), N (16.06%), and O (15.06%).


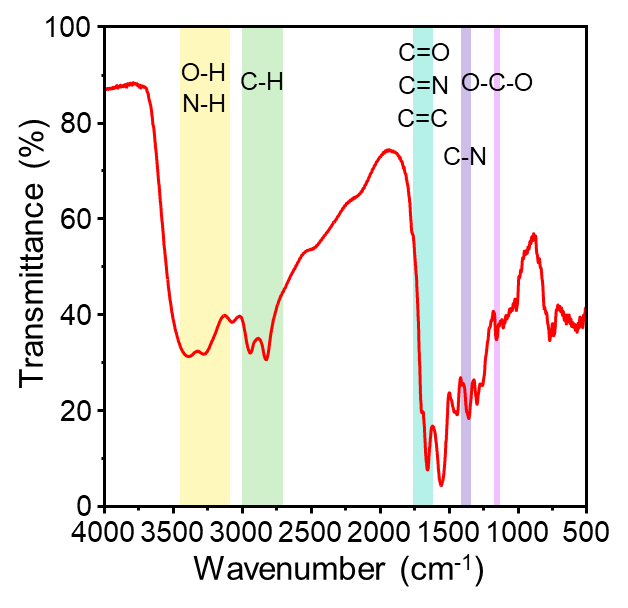


**Figure S4** The FTIR spectrum of the CDs. The FTIR spectrum shows that the CDs exhibit stretching vibrations corresponding to O–H/N–H (3450-3100 cm⁻¹), –CH3/–CH2– (3000-2700 cm⁻¹), C=O/C=N/C=C (1755-1670, 1690-1640, and 1680-1620 cm⁻¹), C–N (1420-1350 cm⁻¹), and O–C–O (1130-1060 cm⁻¹).

**
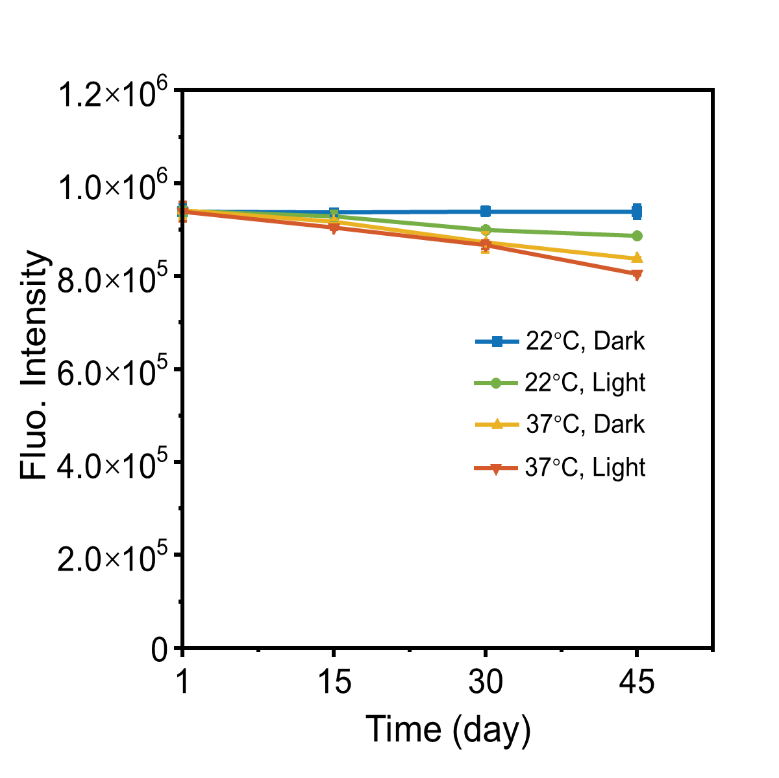
**

**Figure S5** Time course of the fluorescence spectra of the CDs under different temperature and lighting conditions.


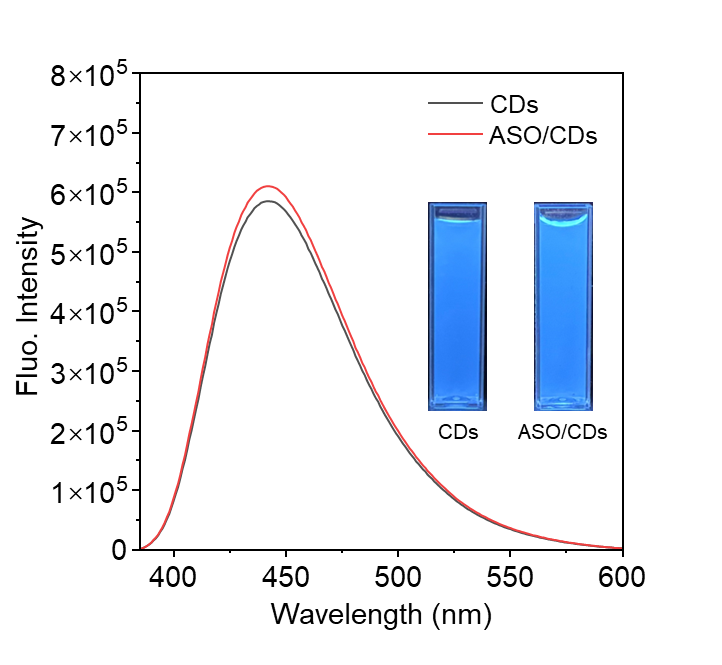


**Figure S6** The fluorescence spectra of the CDs and the ASO/CDs with a CD-to-ASO mass ratio of 20:1. The results show that loading with ASO has a negligible impact to the emission of the CDs. The inset shows the digital photographs of the CDs (left) and the ASO/CDs (right) solutions under 365 nm-UV irradiation.


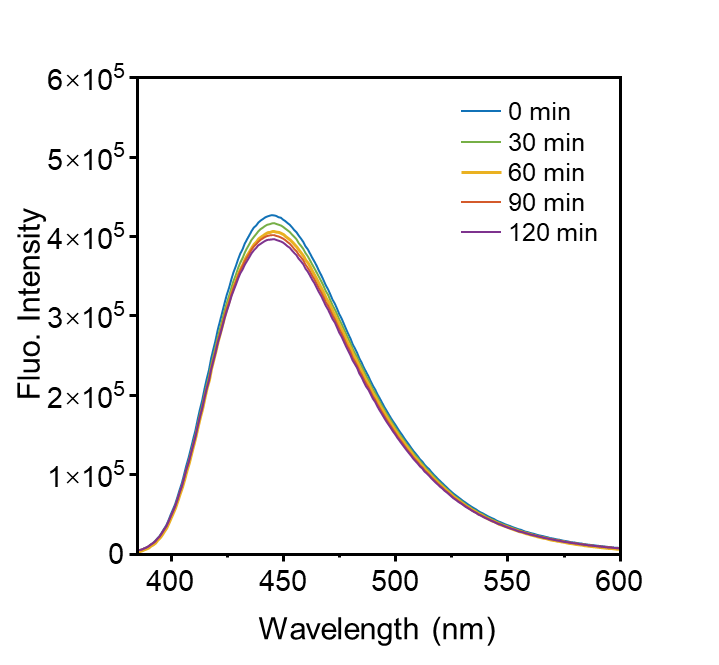


**Figure S7** Fluorescence spectrum of the ASO/CDs with a CD-to-ASO mass ratio of 20:1 in seawater with a salinity of 32‰ at different times. The results indicate good stability of the ASO/CDs in seawater.


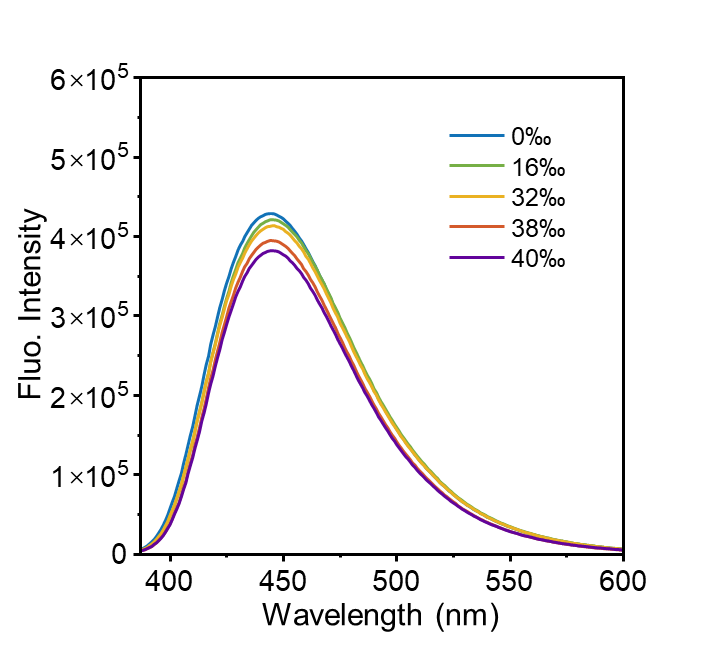


**Figure S8** Fluorescence spectrum of the ASO/CDs with a CD-to-ASO mass ratio of 20:1 in seawater at different salinities. The results indicate that seawater at different salinities has little effect on the stability of ASO/CDs.

**Table S2** Sequence information of the *empA* gene sequence (GenBank: NZ_CP031478.1) of *V. anguillarum*.

| Type | Sequence (5'→3') |
| --- | --- |
| Nucleotide sequence (1836 bp) | ATGAAAAAAGTACAACGTCAAATGAAGTGGCTATTCCTCGCAGCCTCTATCAGCGCAGCACTACCAGTATCAGCCGCGAAAATGGTGCAGGTTGATGATCCAAGTCTACTAGAACAAGCTTTATCCATGCAGGCTCGCAGTATTGTGCCAACACAAAATGGCTTTCAGATGGTAAAAAGCGTCACTCTACCTAATGGTAAAGTCAAAGTCCGTTACCAGCAGATGTACCATGGTTTACCGGTCTTTAATACGTCAGTCGTAGCAACGCAAACTGAGAAAGGTATCGGCCAAGTTTACGGCATGATGGCGCAGCAAATTGATTCTGATGTGGTAAGTACTTCACCACAAGTTGAACAAAAGCAGGCGGTTAGTATTGCACTTACGCATTACCAACAACAAAACCCATCATTGACGAGTGCGGATCTTGTTACAGAAAACGAGCGTGCTCAATTGATGGTGCGTTTGGACGAAAACCAAATAGCTCAAATGGTTTATTTAGTCGACTTCTTTGTTGCAACCAATGAGCCTGCACGGCCATTTTTCTTTATTGATGCGAACTCTGGTGACGTATTACAAACTTGGGAAGGGTTAAACCATGCAGAGGCGACGGGTACTGGACCTGGTGGAAATCAGAAGACAGGTTTTTATCAGTACGGAACAGATTTCCCAGGGTTAGTGATAAATAAAGTAGGCAACACCTGTAGCATGGTGAATAGCGCTGTAAAAACCGTGGATATGAAGCATGCGACATCGGGTGGTTCAACGTTCAGCTATTCATGTACCGACGCGAGTAATTACAACGATTACAAAGCGATTAATGGCGCTTATTCACCACTTAATGATGCACACTACTTTGGTAAAGTGGTGTTCGATATGTACAAAGATTGGATGAACACCACTCCATTAACCTTCCAGCTAACTATGCGTGTGCATTATGATAGCAACTATGAAAACGCCTTCTGGAACGGATCGTCCATGACCTTTGGTGATGGCCAAAATACATTTTATCCATTAGTGGATATCAACGTCAGCGCGCATGAAGTGAGCCACGGTTTTACCGAGCAGAACTCAGGGCTTGTTTACCAAAATATGTCTGGTGGGATGAATGAAGCGTTCTCTGATATTGCTGGGGAAGCAGCTGAATTTTATATGAAAGGCAGCGTTGACTGGGTGGTCGGGAGCGACATTTTCAAATCTTCCGGTGGCTTACGTTATTTTGATCAACCGTCAAAAGATGGCCGTTCTATCGATCACGCTTCTCAATACTACAATGGATTAAATGTTCACTATTCAAGTGGTGTGTTTAATCGCGCGTATTATTTGCTAGCCAACAAAGCCAATTGGTCGGTTCGTAAAGGTTTTGAAGTGTTCACCGTCGCTAACCAGTTATATTGGACAGCAAACAGTACCTTTGACCAAGGTGGATGTGGCGTCGCAAAAGCGGCACAAGACTTAGGTTATAACAAAGCGGATGTGGTTGATGCCTTTAACCAAGTGGGCGTAAATGCTAGCTGTGGCGTAGTGCCTCCAACGGAGAATGTGCTAGAAAAAGGCAAACCAGTAATAGGGTTACAAGGTACGCGAAGCTCCGAAGCCTTCTATACCTTTACGGTCGCAAGCTCAACCAGCGCTAAAGTTTCGATCAGTTTGGGCTCAGGTGATGCTGATTTGTATGTCAAAGCCGGCAGCAAACCAACCACTTCTTCGTGGGATTGTCGCCCTTACAAATCGGGCAACAATGAACAGTGCACAATTTCTGCAACACCAGGAACCACCTACCATGTGATGCTCAAAGGCTATAGTAACTACAGTGGTGTAACGTTAAGACTGGATTAA |

Note: The ASOs tested in this study were designed from the 337^th^, 1082^sd^, 1569^th^, and 1739^th^ base positions of the *empA* gene sequence.


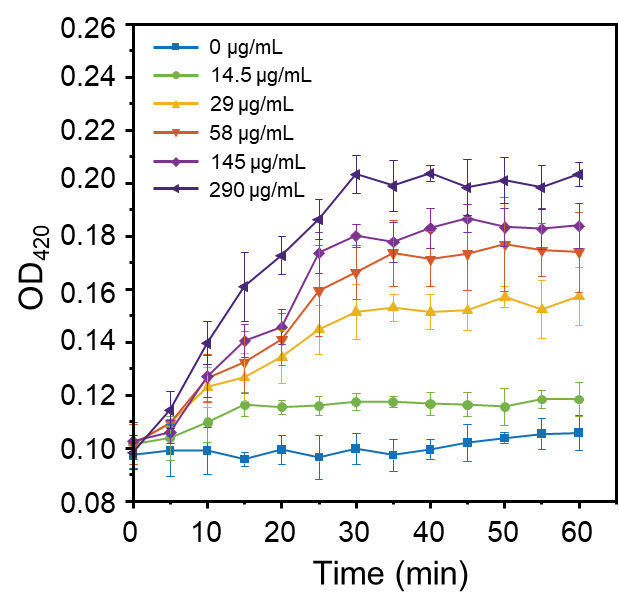


**Figure S9** Time courses of measured OD_420_ for the *V. anguillarum* (200 μL, 10^6^ CFU/mL) treated with the CDs at different concentrations (0, 14.5, 29, 58, 145, and 290 μg/mL) upon addition of ONPG. Note that Increase in OD_420_ implies the increase in the leakage of *β*-galactosidase.


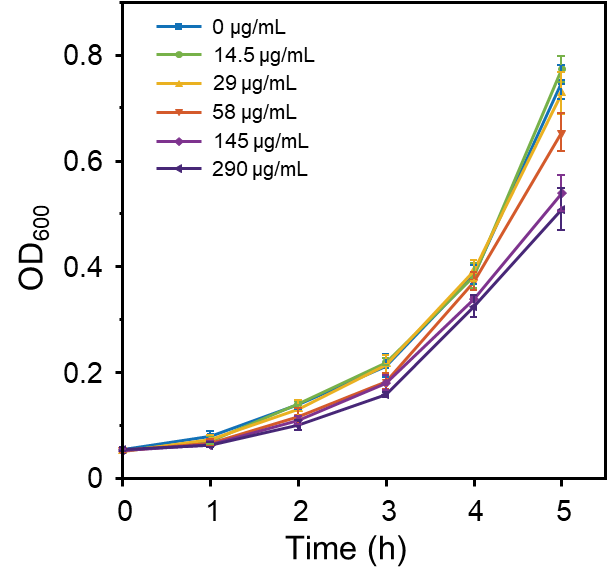


**Figure S10** The growth of the *V. anguillarum* (200 μL, 10^6^ CFU/mL) measured after treatment with different concentrations (0, 14.5, 29, 58, 145, and 290 μg/mL) of the CDs for different times (0, 1, 2, 3, 4 and 5 h).


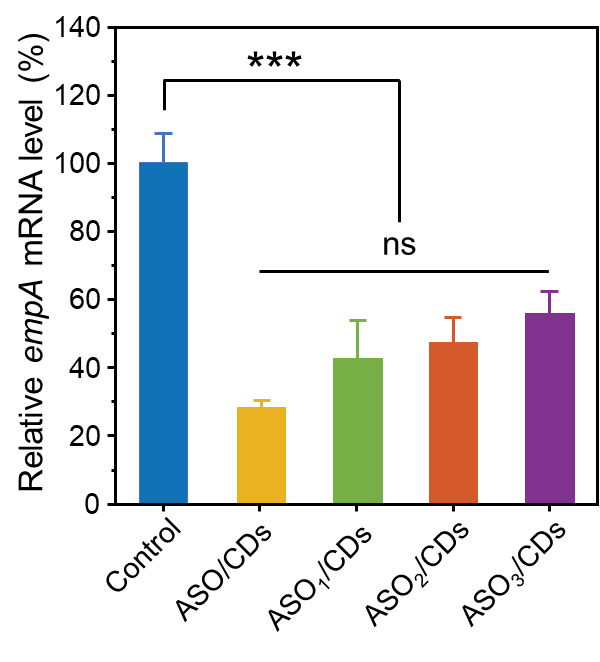


**Figure S11** Histogram of the transcription levels of the *epmA* gene in the *V. anguillarum* upon treatment for 2 h with water (Control), ASO/CDs, ASO_1_/CDs, ASO_2_/CDs, and ASO_3_/CDs. Note that ASO, ASO_1_, ASO_2_, and ASO_3_ were designed from the 250^th^, 80^th^, 737^th^, and 1481^st^ base positions of the *empA* gene sequence, respectively.


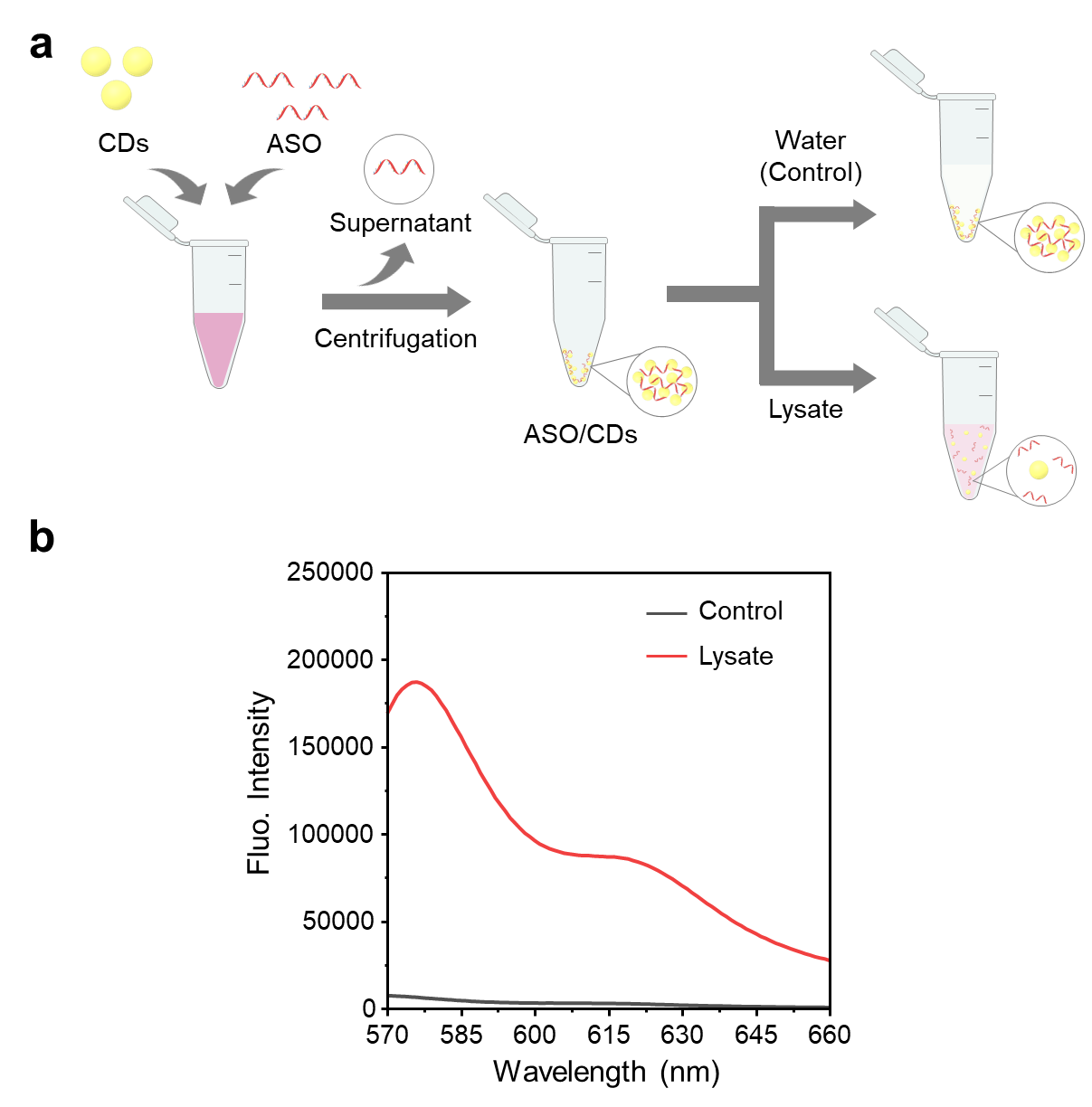


**Figure S12** Test of the release of the ASO from the CDs. (a) Schematic illustration for the procedure of probing the release of the Cy3-ASO from the CDs upon treatment of the Cy3-ASO/CDs with the lysate of *V. anguillarum*. The Cy3-ASO/CDs treated with water was used as the control. (b) Fluorescence spectrum of the supernatant of the Cy3-ASO/CDs (Cy3-ASO:CDs = 1:5, mass ratio) upon treatment with *V. anguillarum* lysate for 1 h. The Cy3-ASO/CDs treated with water were used as the control. Note that the concentrations of the Cy3-ASO and the mixing ratio to CDs were consistent to the optimized results obtained from Figure 2c.


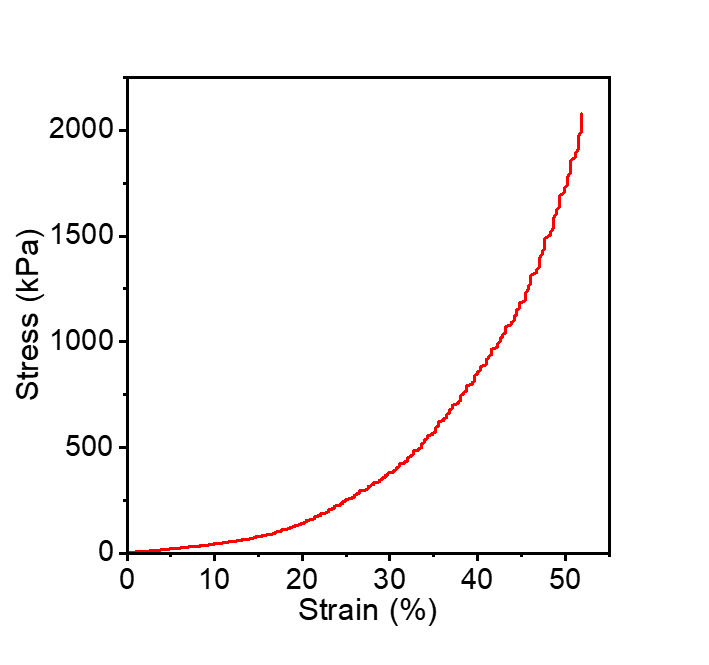


**Figure S13** The stress-strain curve of hydrogel at 50% compression.


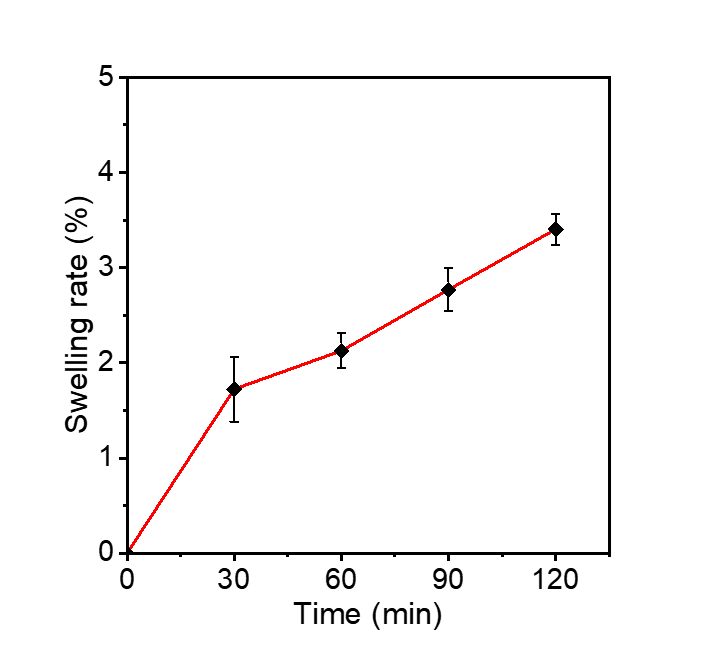


**Figure S14** Line chart of the swelling ratio of the hydrogel after incubation for different times in seawater.


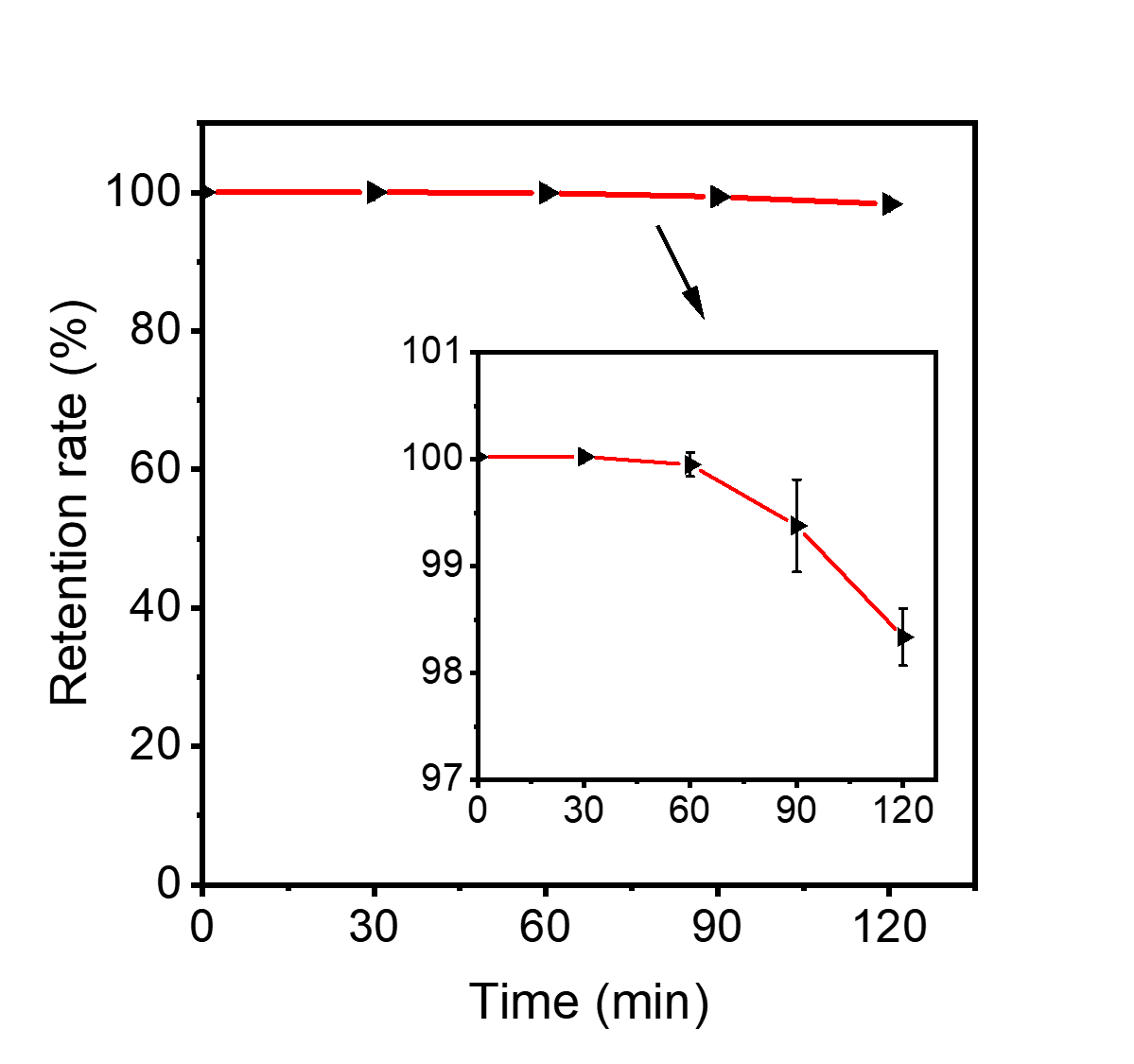


**Figure S15** The retention rate of the CDs at a turbot wound site sealed by the hydrogel for different times in seawater.

**Table S3** The morbidity and mortality of the turbots from various groups.

| Various groups | Number of incidences and deaths at different time points (fish) | | | | | | | | Total number (fish) | Morbidity / mortality (%) |
| --- | --- | --- | --- | --- | --- | --- | --- | --- | --- | --- |
|  |  | 1 day | 2 day | 3 day | 4 day | 5 day | 6 day | 7 day |  |  |
| Uninfected | Incidence | 0 | 0 | 0 | 0 | 0 | 0 | 0 | 0 | 0 (morbidity) |
|  | Death | 0 | 0 | 0 | 0 | 0 | 0 | 0 | 0 | 0 (mortality) |
| Control | Incidence | 0 | 1 | 3 | 6 | 9 | 9 | 10 | 10 | 100 (morbidity) |
|  | Death | 0 | 0 | 0 | 2 | 3 | 5 | 6 | 6 | 60 (mortality) |
| CDs | Incidence | 0 | 0 | 2 | 4 | 9 | 9 | 9 | 90 | 90 (morbidity) |
|  | Death | 0 | 0 | 0 | 1 | 3 | 4 | 4 | 4 | 40 (mortality) |
| ASO | Incidence | 0 | 0 | 2 | 5 | 8 | 9 | 10 | 100 | 100 (morbidity) |
|  | Death | 0 | 0 | 1 | 2 | 3 | 5 | 5 | 5 | 50 (mortality) |
| ASO/CDs | Incidence | 0 | 0 | 2 | 6 | 8 | 9 | 9 | 90 | 90 (morbidity) |
|  | Death | 0 | 0 | 0 | 2 | 3 | 4 | 4 | 4 | 40 (mortality) |
| Hydrogel | Incidence | 0 | 0 | 2 | 5 | 9 | 9 | 10 | 10 | 100 (morbidity) |
|  | Death | 0 | 0 | 1 | 3 | 3 | 5 | 5 | 5 | 50 (mortality) |
| ASO/CDs- hydrogel composite | Incidence | 0 | 0 | 0 | 0 | 0 | 0 | 0 | 0 | 0 (morbidity) |
|  | Death | 0 | 0 | 0 | 0 | 0 | 0 | 0 | 0 | 0 (mortality) |
| Oxolinic acid | Incidence | 0 | 0 | 0 | 0 | 0 | 0 | 0 | 0 | 0 (morbidity) |
|  | Death | 0 | 0 | 0 | 0 | 0 | 0 | 0 | 0 | 0 (mortality) |


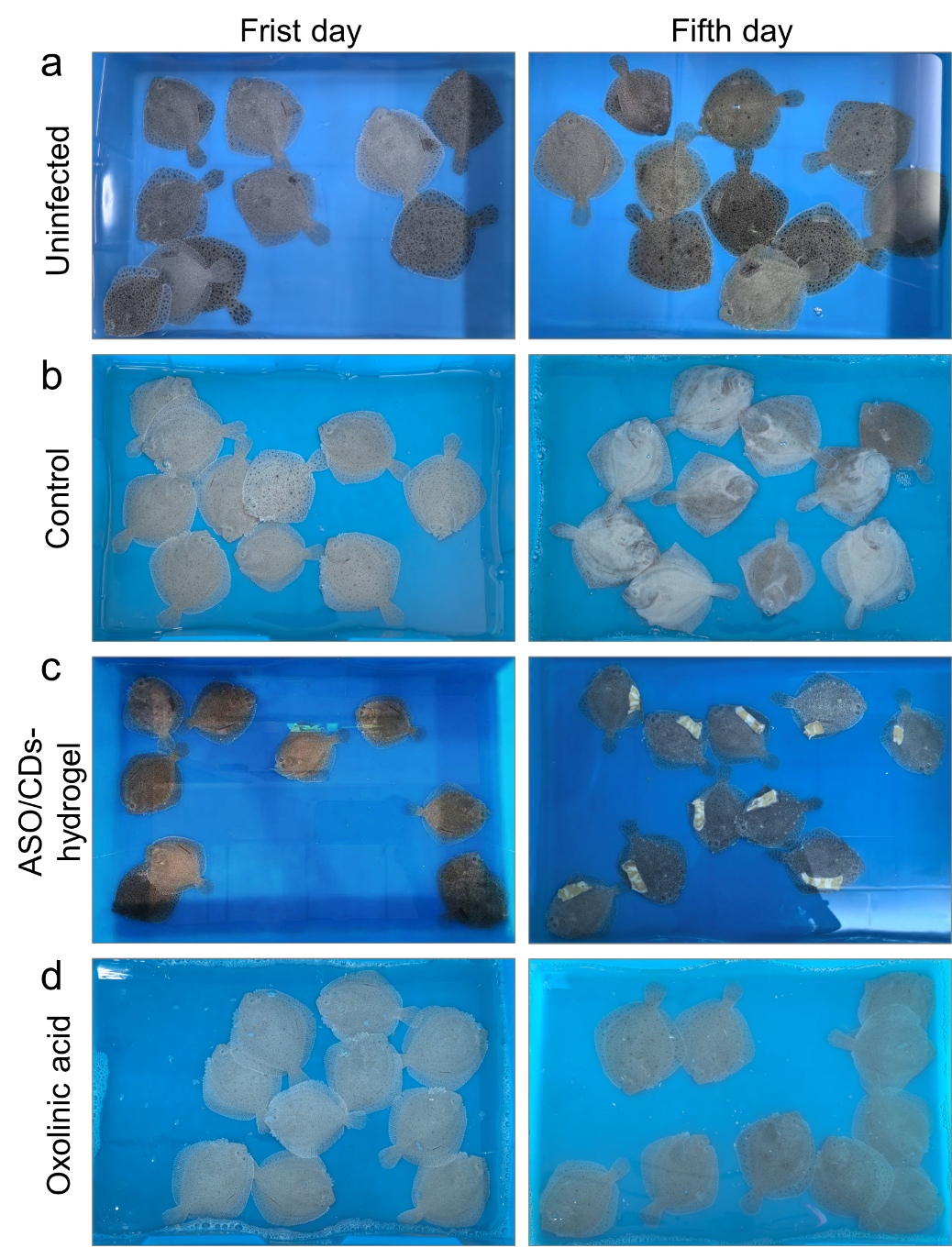


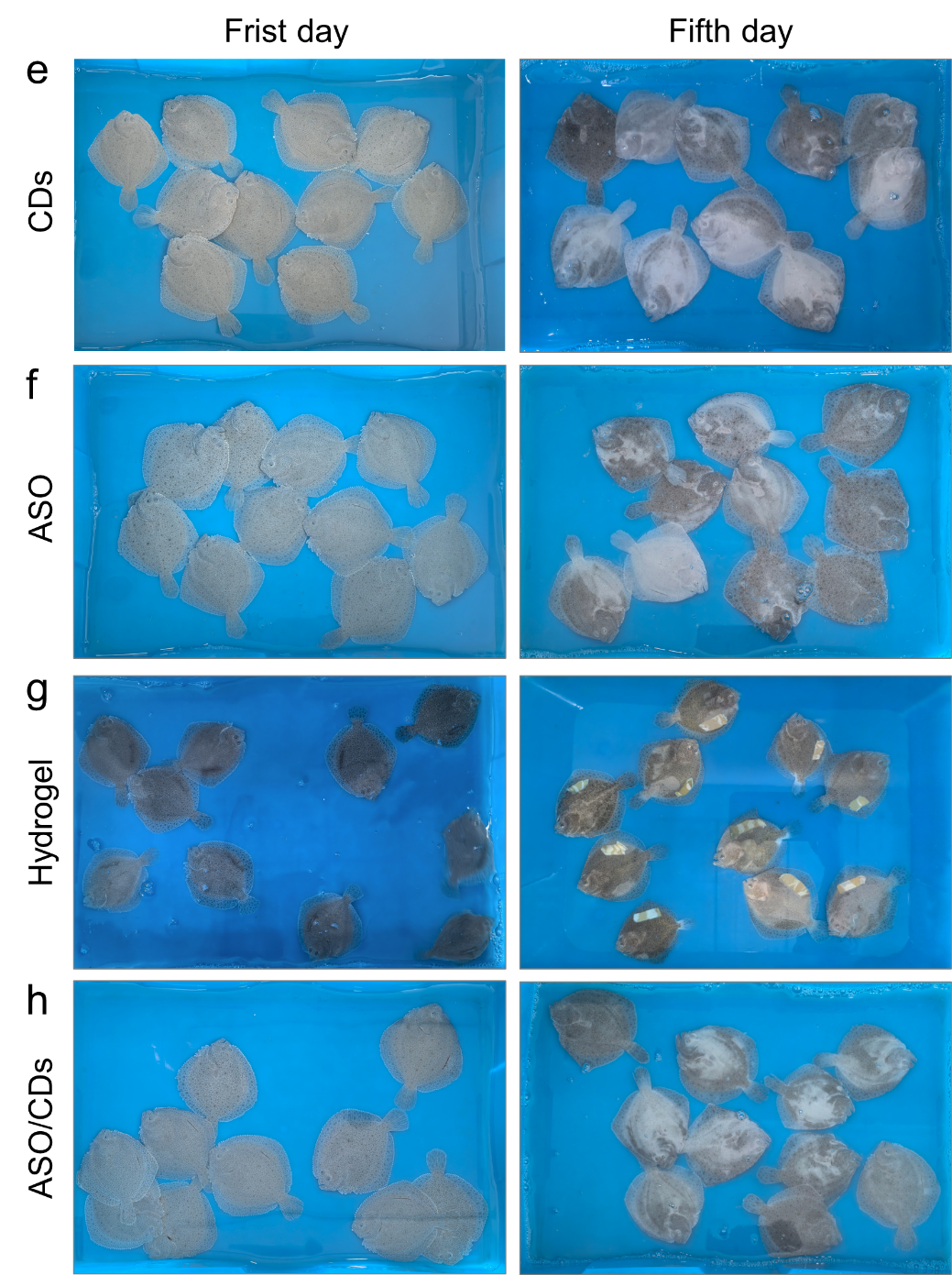


**Figure S16** Representative photographs of the scratched turbots, as captured one day and five days after scratching. The eight groups of the turbots include uninfected turbots (a), infected turbots treated with water (Control) (b), the ASO/CDs-hydrogel composite (c), oxolinic acid (d), the CDs (e), the ASO (f), the hydrogel (g), the ASO/CDs (h).


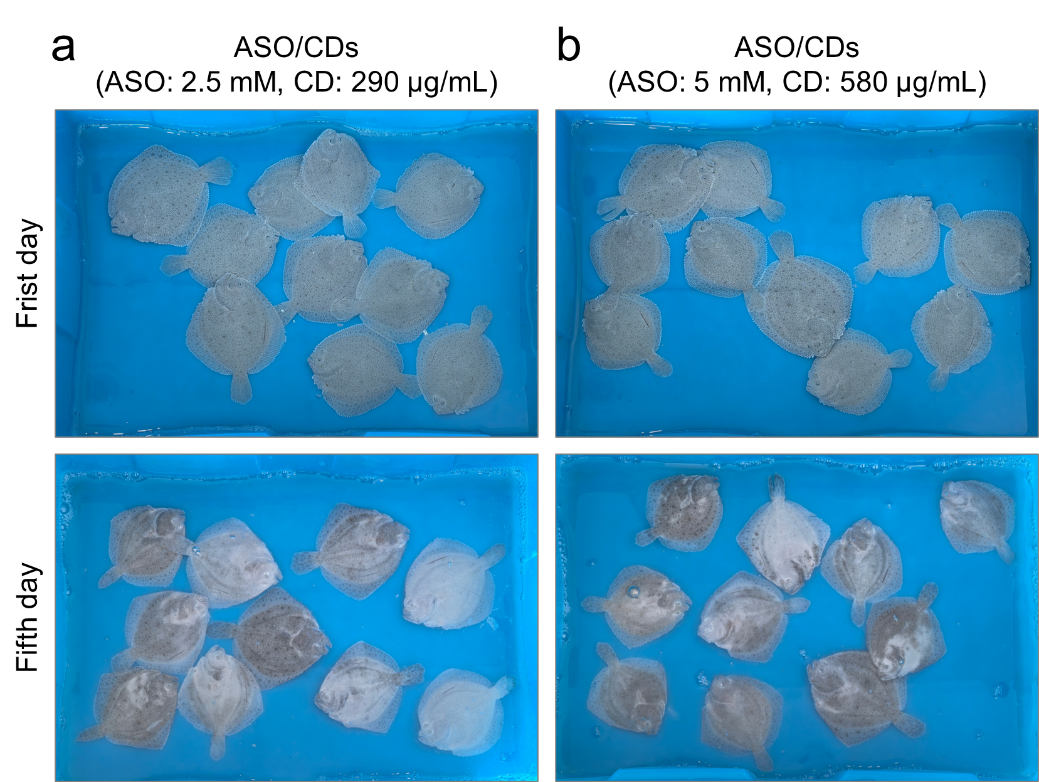


**Figure S17** Representative photographs of the scratched turbots captured one day and five days after scratching. The two groups of the infected turbots were treated with different concentrations of the ASO/CDs, which are higher than that of the ASO/CDs sealed with the hydrogel by five and ten times.


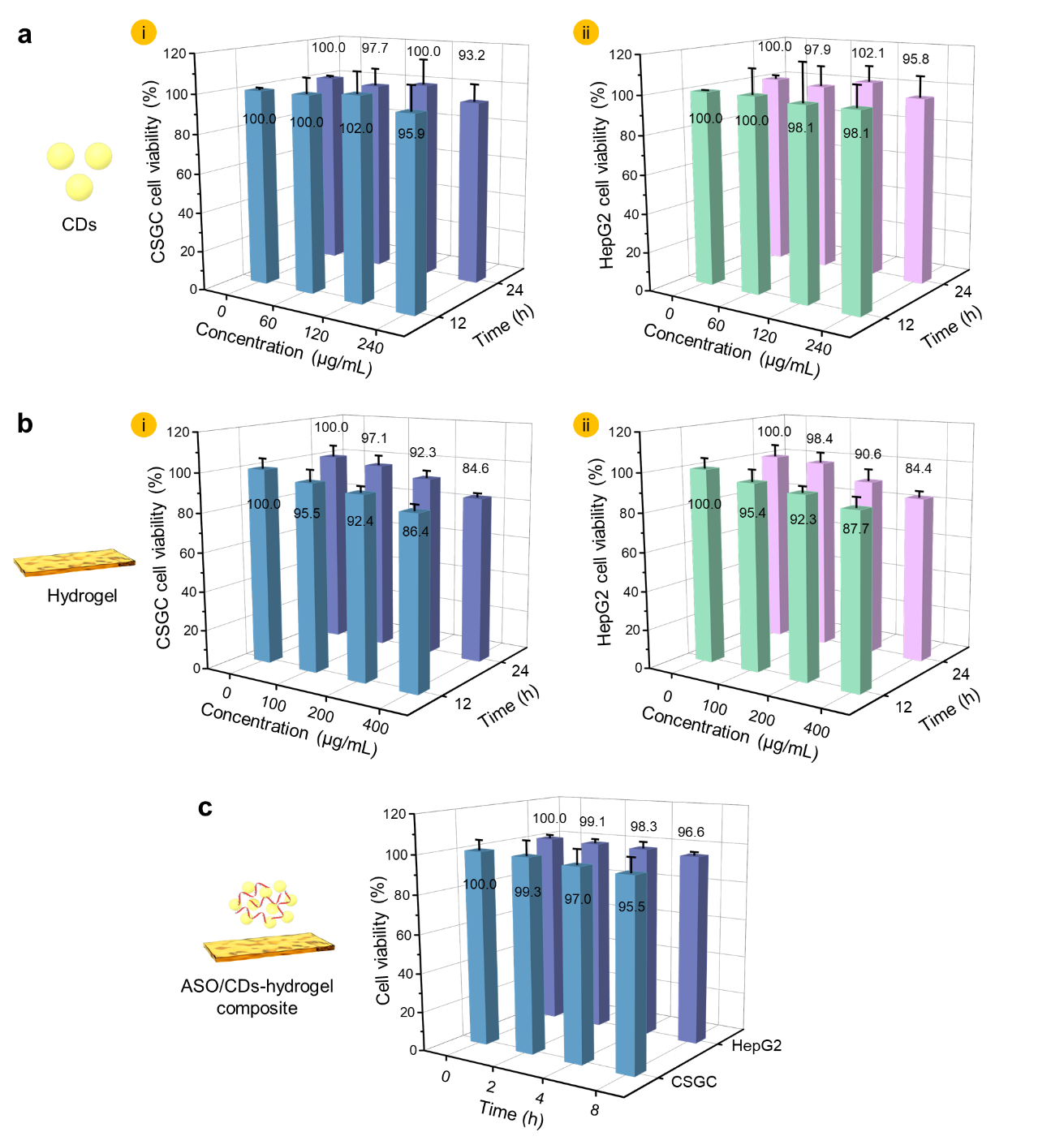


**Figure S18** The cytotoxicity of the CDs, the hydrogel, and the ASO/CDs-hydrogel composite. (a) The cell viabilities of CSGC (i) and HepG2 cells (ii) (100 μL and 5×10^4^ cell/mL, both) treated with different concentrations (0, 60, 120, and 240 μg/mL) of the CDs for different times (12 and 24 h). (b) The cell viabilities of CSGC (i) and HepG2 cells (ii) (100 μL, 5×10^4^ cell/mL) treated with different concentrations (0, 60, 120, and 240 μg/mL) of the hydrogel for different times (12 and 24 h). (b) The cell viabilities of CSGC (i) and HepG2 cells (ii) (100 μL, 5×10^4^ cell/mL) treated with the ASO/CDs-hydrogel composite for different times (0, 2, 4, and 8 h).
